# Antitumor-Relevant Evaluation of Liquid and Microencapsulated *Lepidium meyenii* and *Bactris gasipaes* Extracts Reveals Species- and Formulation-Dependent Activity

**DOI:** 10.3390/molecules31183286

**Published:** 2026-09-16

**Authors:** Fabián Castillo-Solís, Juan A. Puente-Pineda, Diana Celi, Henrry M. Osorio, Elena Coyago-Cruz, Eduardo Tejera, Orestes Lopez, Rebeca Gonzalez-Pastor

**Affiliations:** 1 Centro de Investigación Biomédica (CENBIO), Facultad de Ciencias de la Salud Eugenio Espejo, Universidad UTE, Quito 170527, Ecuador; fabianc.castillo@ute.edu.ec (F.C.-S.); juan.puente@ute.edu.ec (J.A.P.-P.); 2 Facultad de Ciencia e Ingeniería en Alimentos y Biotecnología, Universidad Técnica de Ambato, Ambato 180104, Ecuador; od.lopez@uta.edu.ec; 3 Facultad de Ingeniería y Ciencias Aplicadas, Carrera de Ingeniería en Agroindustria, Universidad de Las Américas, Quito 170504, Ecuador; diana.celi@udla.edu.ec; 4 Facultad de Ingeniería y Ciencias Aplicadas, Carrera Ingeniería en Biotecnología, Universidad de Las Américas, Quito 170504, Ecuador; eduardo.tejera@udla.edu.ec; 5 Departamento de Física, Escuela Politécnica Nacional, Av. Ladrón de Guevara E11-253, Quito 170525, Ecuador; henrry.osorio@epn.edu.ec; 6 Carrera de Ingeniería en Biotecnología de los Recursos Naturales, Universidad Politécnica Salesiana, Sede Quito, Campus El Girón, Av. 12 de Octubre N2422 y Wilson, Quito 170143, Ecuador; ecoyagoc@ups.edu.ec; 7 Bio-Cheminformatics Research Group, Facultad de Ingeniería y Ciencias Aplicadas, Universidad de Las Américas, Quito 170504, Ecuador

**Keywords:** maca, chonta, *Lepidium meyenii*, *Bactris gasipaes*, microencapsulation, spray drying, antitumor activity, inflammation-related response, lepidiline alkaloids, tumor-cell selectivity

## Abstract

*Lepidium meyenii* and *Bactris gasipaes* are traditionally consumed plant foods with documented antioxidant and inflammation-related activities and emerging evidence of cancer-related biological effects. Accordingly, we compared the antitumor-relevant activity of these chemically distinct extracts and evaluated the effect of microencapsulation. Liquid extracts were chemically characterized, spray-dried with maltodextrin- or maltodextrin–gum arabic, and evaluated in tumor and non-tumor cell models, with antioxidant and inflammation-related endpoints as complementary responses; *L. meyenii* was further examined by bioactivity-guided fractionation. Chemical characterization confirmed that *L. meyenii* was enriched in organic acids, phenolics, and alkaloids, whereas *B. gasipaes* was dominated by carotenoids. *B. gasipaes* liquid extract showed lower IC_50_ values (0.27–0.63 mg/mL), whereas *L. meyenii* liquid extract showed higher calculated therapeutic indices (2.28–3.64). Both extracts showed encapsulation efficiencies above 97%, with microencapsulation generally reducing antiproliferative potency. Bioactivity-guided fractionation associated the strongest tumor-cell responses with *L. meyenii* fractions containing signals putatively annotated as lepidilines A, B, and D. Additionally, *L. meyenii* extract reduced LPS-induced nitric oxide production without reproducing a broad LPS-like transcriptional response. These findings support further studies to evaluate enriched constituents, clarify mechanisms across broader cell models, and explore spray-dried carrier behavior during storage and reconstitution.

## 1. Introduction

Plant extracts are chemically complex matrices whose phytochemical profile and biological activity are influenced by the plant material and the conditions applied during extraction, processing, and storage [1]. Plant extracts and extract-derived compounds continue to be investigated as complex bioactive systems capable of modulating cancer-related processes such as proliferation, apoptosis, oxidative stress, inflammation, angiogenesis, and cell migration [2,3,4]. Together, these considerations justify the comparative evaluation of edible plant matrices with documented traditional use, chemical diversity, and bioactivity relevant to tumor-cell models. Nevertheless, how matrix-specific chemical profiles interact with microencapsulation to influence biological response and tumor selectivity remains insufficiently understood.

In this context, maca (*Lepidium meyenii*) and chonta (*Bactris gasipaes*) represent two Latin American edible plant matrices with distinct phytochemical profiles and reported biological properties. *L. meyenii*, an Andean crop traditionally consumed as a food and used for its nutritional, energizing, and reproductive-health-related properties, contains several classes of secondary metabolites, including macamides, macaenes, glucosinolates, alkaloids, phenolic compounds, and polysaccharides [5,6]. Previous experimental studies have reported antioxidant, anti-inflammatory, and cancer-cell-related effects for *L. meyenii* extracts, fractions, or isolated constituents, including chemically characterized fractions enriched in macamides, macaenes, or glucosinolates [7,8,9]. At the same time, reported effects vary with extract composition, fraction or compound evaluated, and experimental model, supporting a context-specific assessment of its biological potential [5,10]. *B. gasipaes* is an Amazonian palm species valued as a food resource, with ethnopharmacological reports describing uses related to inflammation, infections, fertility, and pain [11]. Its fruit is characterized by carotenoids, phenolic compounds, and lipid-associated bioactives, which have been linked experimentally to antioxidant and inflammation-related effects [12,13,14]. Although its direct antitumor activity remains less characterized, this traditional and phytochemical background supports its evaluation as a chemically distinct plant matrix.

One limitation of liquid plant extracts is their susceptibility to degradation during storage, especially when they contain oxidation-sensitive or compositionally diverse metabolites [15,16]. Microencapsulation is widely used to incorporate plant extracts into carrier-based microparticles, improving handling, stability, and protection of bioactive compounds [17,18]. Among the available techniques, spray drying is particularly attractive because it is scalable, cost-effective, and suitable for producing stable powdered ingredients using a broad range of carrier materials [19,20]. Its short exposure times can also help limit thermal degradation of labile compounds, supporting its suitability for the present study [19,21,22]. Maltodextrin and gum arabic are among the most commonly used encapsulating materials in such systems because of their solubility, stabilizing capacity, and compatibility with diverse phytochemical matrices, including phenolic-rich extracts, anthocyanins, carotenoids, oils, flavors, and other labile ingredients. These properties support their use in storage-stable formulations for food, nutraceutical, and ingredient applications [23,24]. Functionally, maltodextrin acts as a water-soluble carrier that favors matrix formation and powder recovery, whereas gum arabic provides emulsifying and interfacial-stabilizing properties useful for systems containing pigment- or lipid-associated constituents [25,26].

Accordingly, *L. meyenii* and *B. gasipaes* were selected as two traditionally consumed edible plant matrices from Andean and Amazonian regions, with contrasting phytochemical profiles, established extraction and formulation conditions, and still incompletely characterized potential relevant to antitumor activity, enabling their evaluation within a common experimental framework. This study aimed to chemically characterize their liquid extracts and to evaluate how spray-drying microencapsulation influenced their biological performance. We hypothesized that the effect of microencapsulation on biological activity and tumor-cell selectivity would also depend on the phytochemical characteristics of each extract. Based on the expected compatibility of the selected carriers with hydrophilic and lipophilic components, *L. meyenii* extract was formulated with maltodextrin, whereas *B. gasipaes* extract was formulated with a maltodextrin–gum arabic matrix, reflecting the predominant chemical features of each extract. Liquid and microencapsulated extracts were compared for antiproliferative activity and tumor-cell selectivity in tumor and non-tumor cell models, with antioxidant and inflammation-related responses evaluated as complementary endpoints. The extract showing the most favorable tumor-cell selectivity profile was further subjected to bioactivity-guided fractionation to identify candidate constituents associated with the observed response.

## 2. Results

### 2.1. Chemical Characterization of Liquid Extracts

Species-specific heat-assisted solvent extraction was used to obtain concentrated liquid extracts suitable for subsequent spray-drying formulation, chemical characterization, and biological evaluation. The extraction conditions were selected based on preliminary optimization studies and adapted to each plant matrix, considering the expected phytochemical profile, its biological relevance, and the influence of solvent, extraction temperature, extraction time, and matrix characteristics on phytochemical recovery [27]. The plant extract preparation process revealed notable differences between the parameters obtained for *L. meyenii* and *B. gasipaes* (Table 1). Under their respective extraction conditions, *L. meyenii* exhibited a higher extraction yield after hydroethanolic extraction, whereas *B. gasipaes* showed a higher concentration of total solids after ethanolic extraction. The values correspond to species-specific extraction protocols, with different solvent concentrations, extraction times, and plant material-to-solvent ratios for each plant matrix.

Following the evaluation of extraction parameters, Table 2 presents the qualitative results of the phytochemical screening of various secondary metabolites obtained from liquid extracts of *L. meyenii* and *B. gasipaes*. The results consistently demonstrated a marked dependence on the solvent systems employed, as well as variability in the presence or absence of specific compounds according to the sample matrix. In this context, *L. meyenii* was characterized by a predominance of water-soluble constituents, whereas *B. gasipaes* exhibited a profile enriched in lipophilic compounds, indicative of a more oil-based extract composition.

Based on the qualitative phytochemical profile, Table 3 shows the concentrations of organic acids, phenolic compounds and alkaloids quantified in *L. meyenii.* None of the targeted organic acids and phenolic compounds were detected in *B. gasipaes* above the applicable analytical detection limits under the conditions used. Thus, malic acid was identified as the predominant organic acid in *L. meyenii*, while 4-hydroxybenzoic acid was the major compound within the phenolic fraction.

In contrast to the compound groups reported for *L. meyenii*, Table 4 shows the carotenoid profile in the liquid extract of *B. gasipaes*. None of the carotenoids were detected in *L. meyenii* above the applicable analytical detection limits under the analytical conditions used. β-Carotene was the predominant carotenoid, followed by β-cryptoxanthin and 9-cis-violaxanthin.

### 2.2. Characterization of Microencapsulated Extracts

#### 2.2.1. Encapsulation Performance

The encapsulation efficiency, yield, and structural conformation of the microencapsulated extracts are shown in Table 5. Encapsulation efficiency was assessed using the main bioactive markers of each extract in the microencapsulated material: polyphenols for *L. meyenii* and β-carotene for *B. gasipaes* (Table S2). Both formulations showed high encapsulation efficiency, with values above 97%, indicating a low proportion of surface-associated bioactive compounds. This was observed despite the use of different encapsulating matrices and the formation of different structural conformations. In both formulations, yields (<67%) remained lower than encapsulation efficiencies, with a slightly higher value for the *B. gasipaes* microencapsulated extract.

#### 2.2.2. Fourier Transform Infrared Spectroscopy (FTIR) Analysis of Liquid and Microencapsulated Extracts

**Figure 1:**
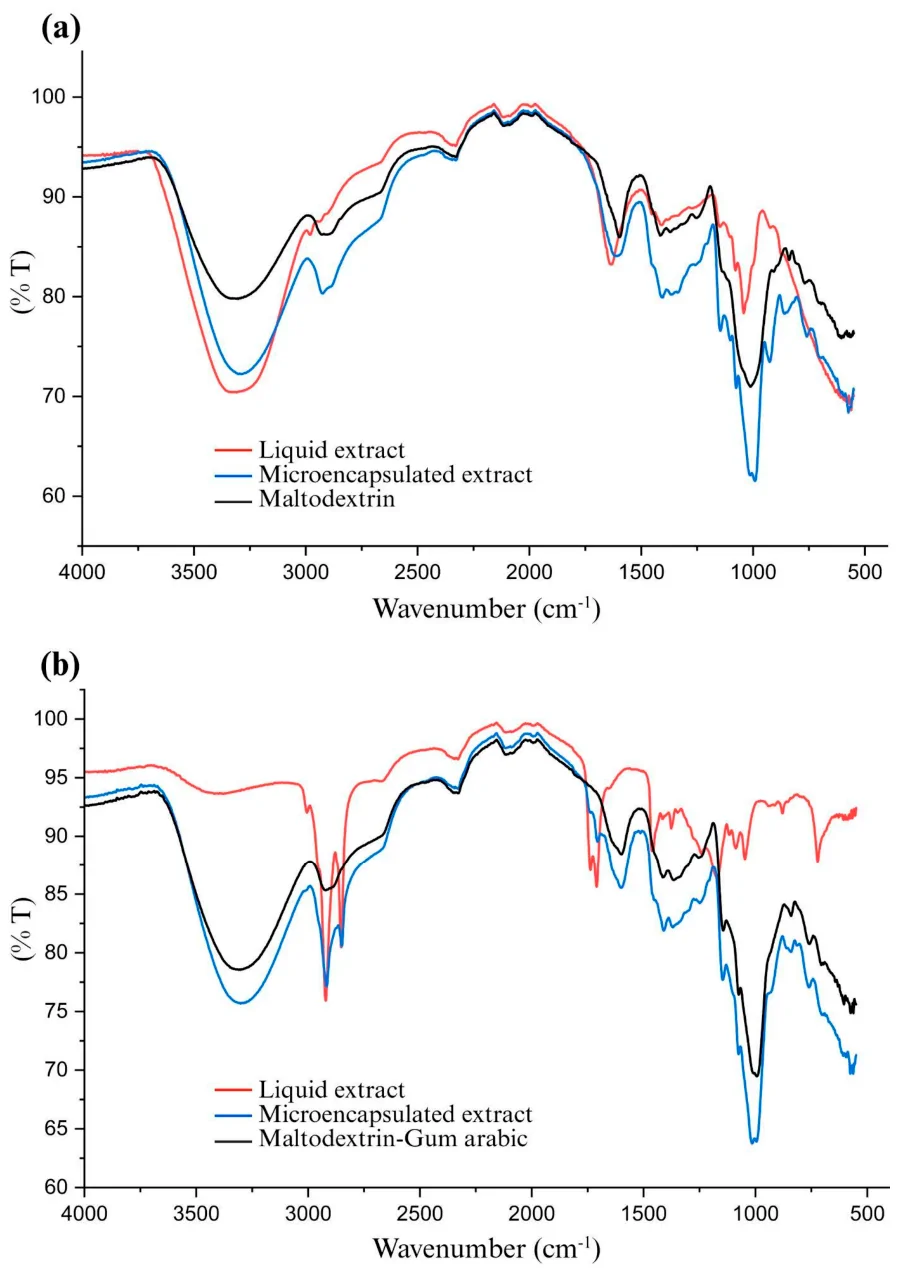
Representative FTIR spectra of liquid and microencapsulated extracts of (**a**) *L. meyenii* and (**b**) *B. gasipaes* from two independent spray-drying batches. The red line represents the liquid extract, the blue line the microencapsulated extract, and the black line the encapsulating polymer based on maltodextrin (*L. meyenii*) or a mixture of maltodextrin and gum arabic (*B. gasipaes*).

According to the FTIR spectra obtained for *L. meyenii* (Figure 1a), the liquid extract, the microencapsulated extract, and maltodextrin showed absorption bands in the 3309–3302 cm^−1^ region, generally associated with O–H stretching vibrations from different hydroxyl-containing components [28,29,30]. Most of the characteristic bands of the liquid extract were not observed after spray drying; however, a characteristic band of the liquid extract at 1409 cm^−1^ was preserved, with a slight shift to 1407 cm^−1^ [31,32,33]. Additionally, the microencapsulated extract showed bands at 2924, 1018, and 927 cm^−1^, which are commonly associated with carbohydrate-based polymer matrices and were consistent with the spectral pattern of maltodextrin at 2925, 990, and 928 cm^−1^, respectively [30,34,35].

For *B. gasipaes* (Figure 1b), several absorption bands present in the liquid extract were not observed after spray-drying, while representative bands at 2922 and 2853 cm^−1^, generally associated with aliphatic groups from lipophilic compounds, were retained in the microencapsulated extract, although with slight shifts to 2920 and 2852 cm^−1^, respectively [36,37]. Additionally, the encapsulated sample exhibited bands at 3319, 1601, and 1017 cm^−1^, associated with functional groups commonly found in polysaccharide-based matrices, which were consistent with the spectral pattern of the maltodextrin–gum arabic encapsulating matrix at 3309, 1599, and 993 cm^−1^, respectively [34,38,39].

The results on efficiency in Table 5 and the modifications observed in the spectral patterns of the liquid extracts, together with the presence of polymer-associated bands in the microencapsulated extracts, supported the formation of microencapsulated structures and the incorporation of both extracts into their respective encapsulating matrices [28,40]. However, the preservation of certain extract-related bands suggested that characteristic extract components remained detectable at or near the particle surface [41].

#### 2.2.3. Structural Features of Reconstituted Microencapsulated Extracts

**Figure 2:**
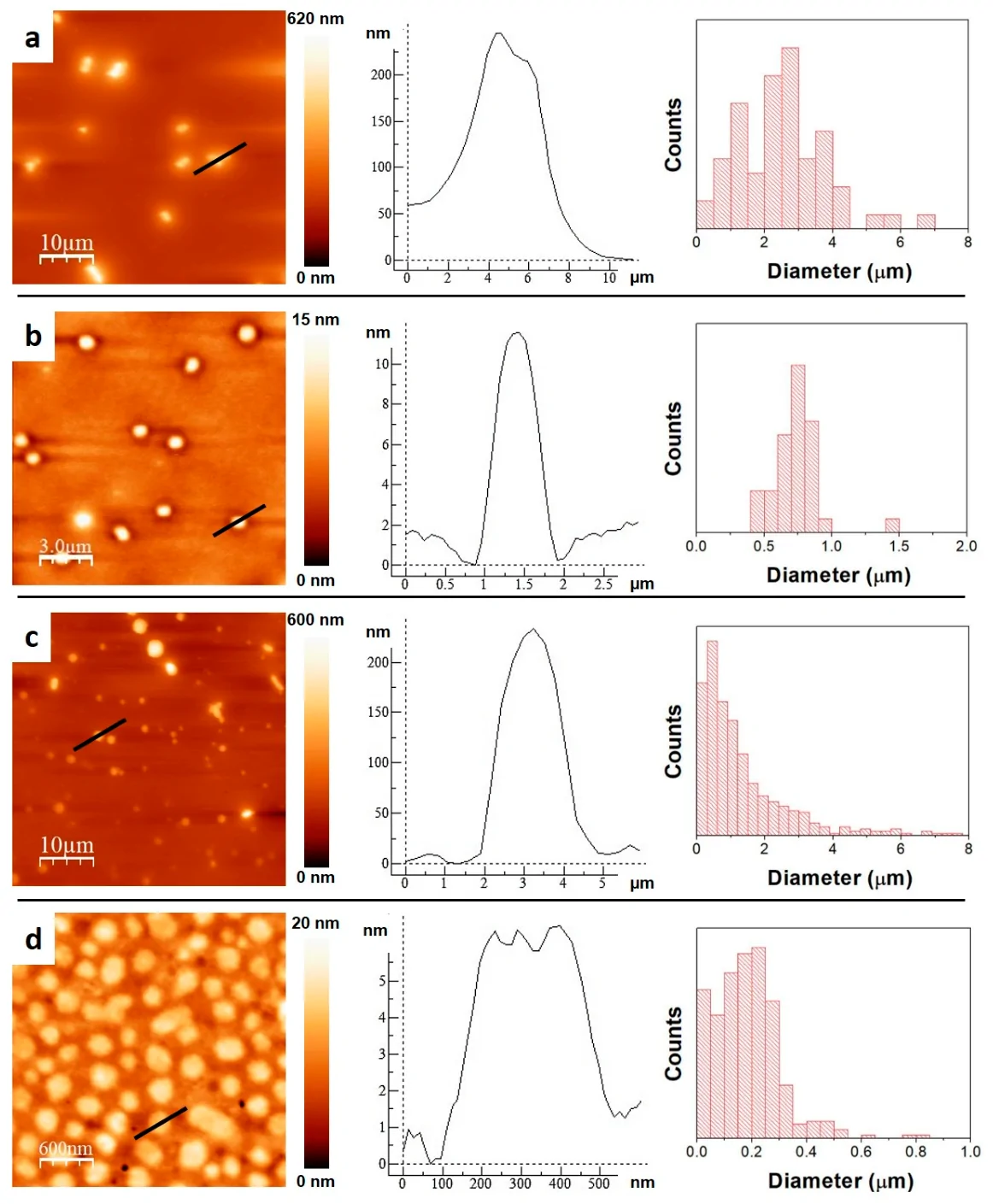
AFM analysis of structures formed after aqueous reconstitution of the spray-dried *L. meyenii* (**a**,**b**) and *B. gasipaes* (**c**,**d**) deposited onto a mica substrate. (**Left**) AFM images. (**Center**) Surface roughness of the observed structures. (**Right**) Particle size distribution of the observed structures.

AFM was used to investigate the morphology and size distribution of structures formed after aqueous reconstitution of the obtained microencapsulated extracts, corresponding to the dispersed state evaluated in the biological assays (Figure 2). Figure 2a shows that the *L. meyenii* formulation formed aggregates with smooth surfaces and a diameter of at least 2.5 μm. However, the magnification presented in Figure 2b shows individual structures with a spherical-like shape and smooth surfaces. The diameter of these structures was in the 0.5–1.0 μm range. On the other hand, Figure 2c,d show that reconstituted *B. gasipaes* formed both large and small structures with a spherical-like shape. Figure 2c shows the presence of larger structures with diameters up to several micrometers. However, the higher-magnification image presented in Figure 2d shows individual submicrometric structures with a spherical-like shape, wrinkled surface, and diameters ranging from 0.05 to 0.4 μm. This wrinkled surface, related to the formation of central voids, was consistent with surface features previously reported for spray-dried carotenoid-containing microcapsules, particularly β-carotene systems, where wrinkling and central void formation have been associated with particle expansion during drying [42,43,44,45]. Overall, after aqueous reconstitution, both formulations retained detectable particulate or aggregated structures, with differences in size range and surface features between *L. meyenii* and *B. gasipaes*. Most of the structures obtained from *L. meyenii* (Figure 2b) and *B. gasipaes* (Figure 2d) formulations did not present a significant incidence of cracks or fissures on the outer surface, indicating a resistant external physical structure.

### 2.3. Evaluation of Antioxidant Activity of L. meyenii and B. gasipaes

The antioxidant activity of *L. meyenii* and *B. gasipaes* extracts was evaluated using DPPH and ABTS assays. DPPH is commonly used to assess radical scavenging in organic media but can be affected by sample solubility or precipitation, whereas ABTS is more adaptable to aqueous and organic systems and can detect both hydrophilic and lipophilic antioxidant responses. Both extracts exhibited low antioxidant activity (Table 6). *L. meyenii* showed weak DPPH radical-scavenging activity and did not reach 50% inhibition within the tested concentration range. Nonlinear regression yielded a model-estimated IC_50_ (Table 6), whereas *B. gasipaes* did not reach 50% inhibition; ABTS activity was negligible for both extracts. Detailed dose–response curves, solvent-specific observations, and ABTS calibration data are provided in Supplementary Materials.

### 2.4. Evaluation of Antiproliferative Activity of Liquid and Microencapsulated Extracts

**Figure 3:**
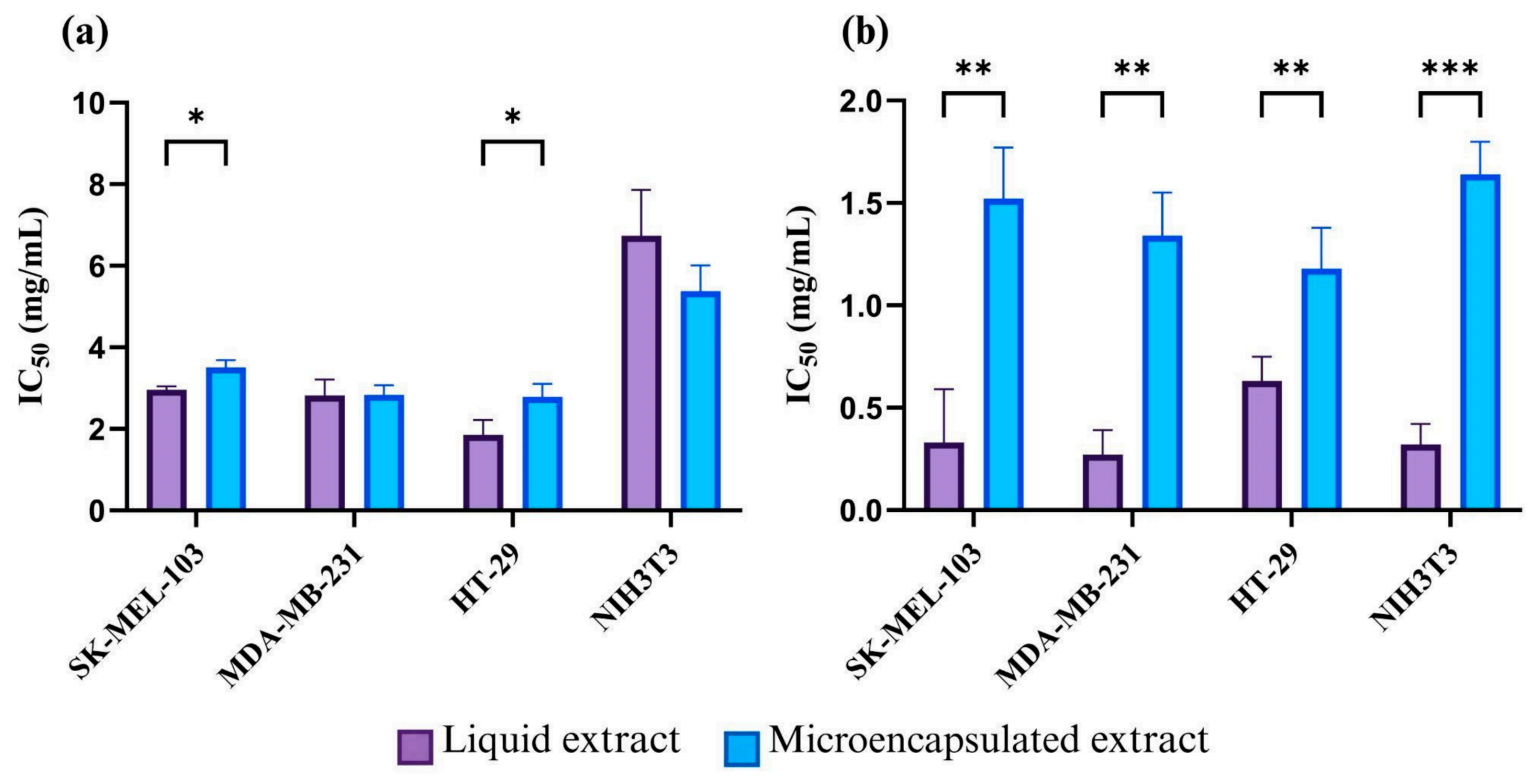
Half-maximal inhibitory concentration (IC_50_) of liquid and microencapsulated extracts after 72 h of treatment. IC_50_ values are expressed as total solids concentration for (**a**) *L. meyenii* and (**b**) *B. gasipaes*, respectively. Purple bars represent the liquid extracts, while blue bars correspond to their microencapsulated versions. The cell lines used were SK-MEL-103 (melanoma), MDA-MB-231 (breast cancer), HT-29 (colon cancer), and NIH3T3 (non-tumorigenic murine fibroblasts). Data are presented as mean ± SD from four independent experiments (n = 4), * *p* ≤ 0.05, ** *p* ≤ 0.01, *** *p* ≤ 0.001.

The antiproliferative activity of liquid and microencapsulated extracts was evaluated by the MTT assay in three tumor cell lines, SK-MEL-103 (melanoma), MDA-MB-231 (breast adenocarcinoma), and HT-29 (colorectal adenocarcinoma), and in the non-tumor NIH3T3 cell line. Liquid extracts were evaluated up to 5 mg/mL of total solids, whereas microencapsulated samples were evaluated up to 71.5 mg/mL of spray-dried powder, comprising both total solids and encapsulating polymer (Tables S3 and S4). The concentration ranges were selected to provide sufficient inhibitory responses for reliable determination of IC_50_ values, defined as the concentration required to inhibit 50% of cell proliferation. The resulting IC_50_ values are shown in Figure 3 and Table S5, revealing differences between liquid and microencapsulated extracts across the evaluated cell lines. To allow direct comparison with the liquid extracts, IC_50_ values obtained from microencapsulated samples were expressed as the corresponding total solids concentration (Table S6). For *L. meyenii*, the liquid extract showed IC_50_ values within a narrow range across the tumor cell lines, from 1.85 mg/mL in HT-29 to 2.96 mg/mL in SK-MEL-103. After microencapsulation, IC_50_ values shifted to a slightly higher range, from 2.79–2.84 mg/mL in HT-29 and MDA-MB-231 to 3.51 mg/mL in SK-MEL-103. Compared with the liquid extract, microencapsulation significantly increased the IC_50_ values in SK-MEL-103 and HT-29, while IC_50_ values in MDA-MB-231 remained practically unchanged. In the non-tumor NIH3T3 cell line, IC_50_ values were higher than those recorded in tumor cell lines for both formulations, with no significant difference between the liquid and microencapsulated extracts. Although microencapsulation decreased the IC_50_ value, this reduction was not statistically significant. For *B. gasipaes*, the liquid extract showed IC_50_ values within a low concentration range across the tumor cell lines, from 0.27 mg/mL in MDA-MB-231 to 0.63 mg/mL in HT-29. After microencapsulation, IC_50_ values were significantly higher in all tumor cell lines, ranging from 1.18 mg/mL in HT-29 to 1.52 mg/mL in SK-MEL-103. In the NIH3T3 cell line, the IC_50_ value also increased significantly after microencapsulation.

Encapsulating-polymer controls were evaluated at concentrations equivalent to those present in the microencapsulated samples and showed ≤20% inhibition of cell proliferation (Table S7), supporting that the observed antiproliferative effects were not primarily attributable to the polymer matrices. Cisplatin was included as the positive control for cell proliferation inhibition (Table S8). Overall, microencapsulated extracts showed higher IC_50_ values than their corresponding liquid extracts in most tumor cell lines, with the clearest formulation-dependent increase observed for *B. gasipaes*.

**Figure 4:**
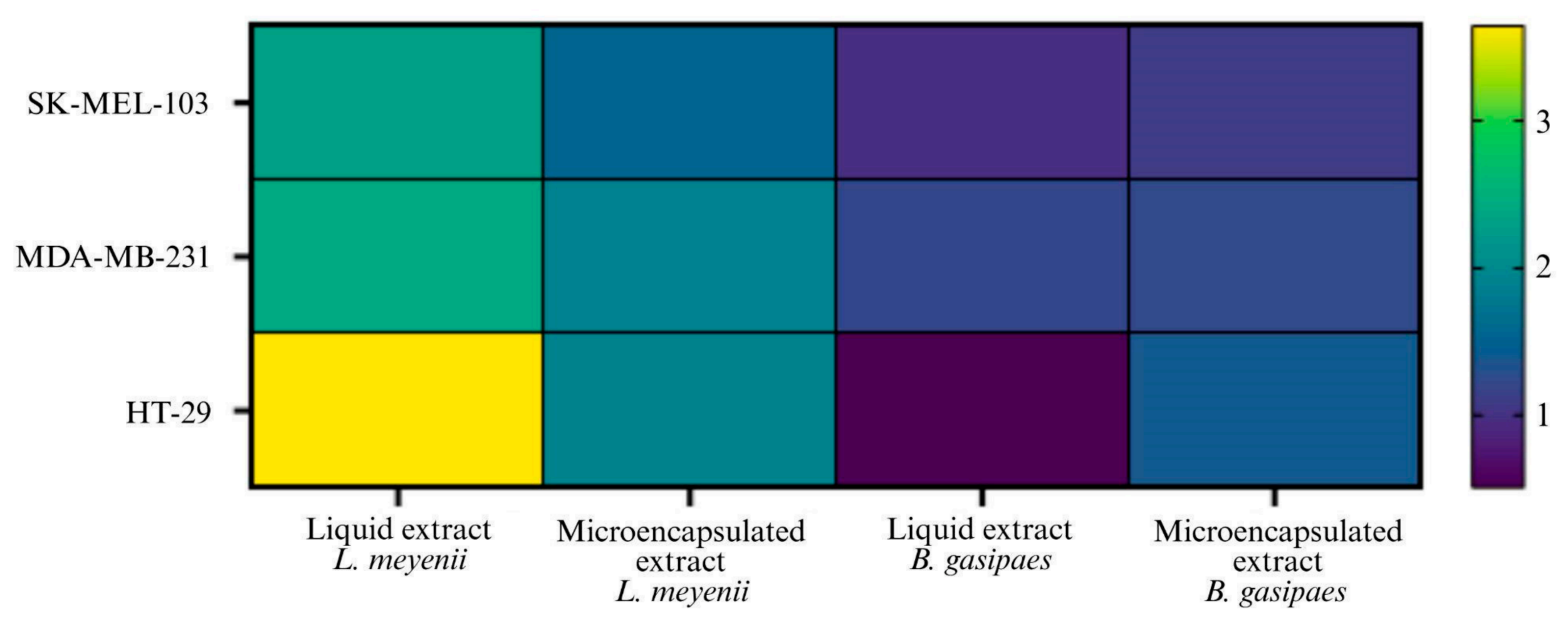
Heatmap of the therapeutic index (TI). TI values for SK-MEL-103 (melanoma), MDA-MB-231 (breast cancer), and HT-29 (colon cancer) cell lines were calculated as the ratio between the IC_50_ values of the non-tumor cell line NIH3T3 and those of the tumor cell lines (IC_50_ non-tumor/IC_50_ tumor), following treatment with liquid and microencapsulated extracts of *L. meyenii* and *B. gasipaes*. The color scale represents the magnitude of the therapeutic index; higher values indicate greater selectivity.

To further evaluate whether the inhibitory effects were preferentially associated with tumor cells, the therapeutic index was calculated using NIH3T3 cells as the non-tumor reference. The therapeutic index (TI) was calculated as the ratio between the IC_50_ values in the non-tumor cell line and the tumor cell lines, where higher values indicated relative selectivity toward tumor cells. As shown in Figure 4, TI values for *L. meyenii* were above 1 in all tumor cell lines for both liquid and microencapsulated extracts. The highest TI was observed for the liquid extract in HT-29 cells, reaching 3.64 based on the extrapolated NIH3T3 IC_50_ value reported in Table S5, which was consistent with the lower IC_50_ observed in this cell line and supported its selection for the subsequent bioactivity-guided fractionation assay. After microencapsulation, TI values for *L. meyenii* remained above 1 but were lower than those of the liquid extract. For *B. gasipaes*, TI values were more variable and were above 1 only in selected conditions, including MDA-MB-231 for both formulations, SK-MEL-103 after microencapsulation, and HT-29 after microencapsulation. Overall, microencapsulation reduced the TI values of *L. meyenii*, whereas its effect on *B. gasipaes* varied among cell lines.

### 2.5. Immunomodulatory and Inflammatory Effects

#### 2.5.1. Anti-Inflammatory Activity in RAW264.7 Macrophages

**Figure 5:**
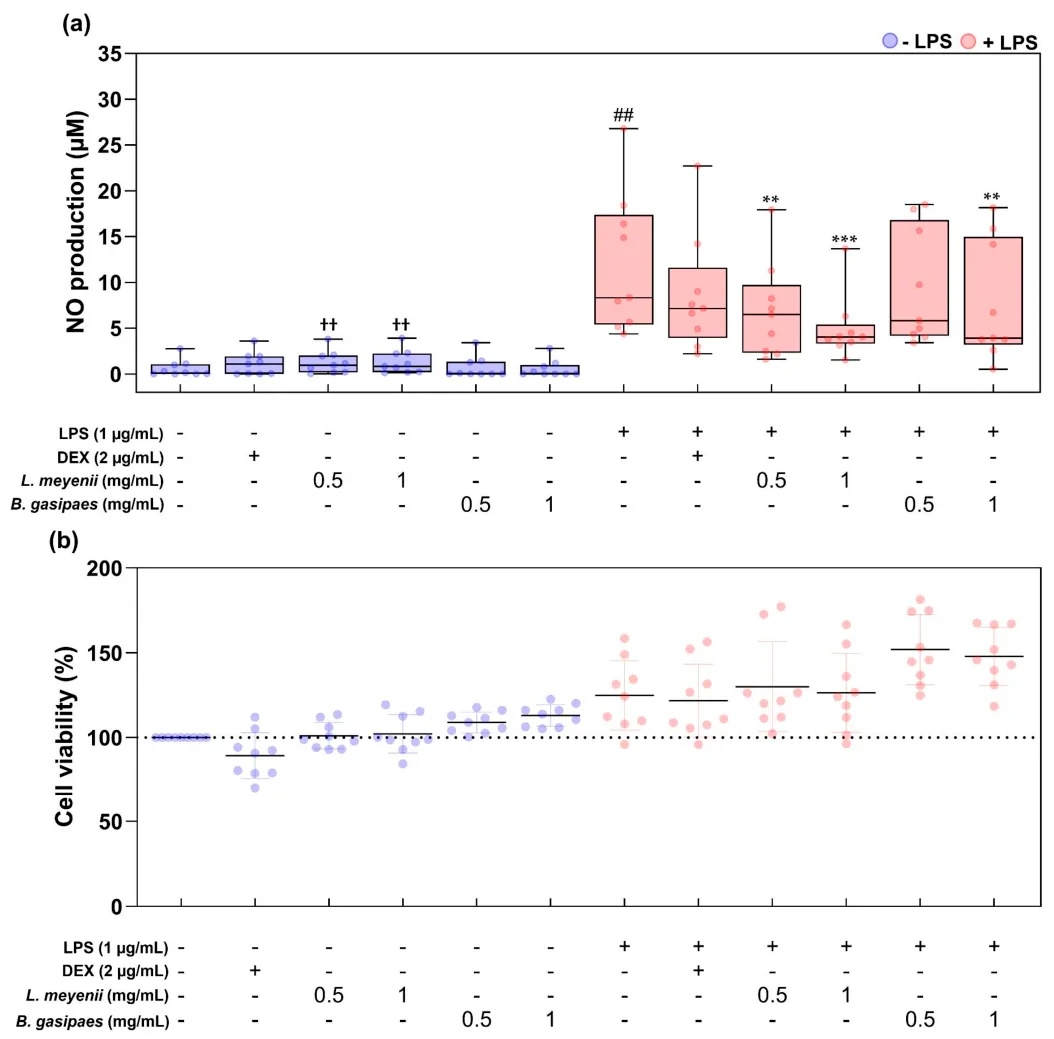
Anti-inflammatory activity and cell viability of *L. meyenii* and *B. gasipaes* extracts in LPS-stimulated cells. (**a**) NO production determined by the Griess assay. Boxplots show the median, interquartile range, and minimum-to-maximum values. (**b**) Relative cell viability determined by crystal violet staining; lines and error bars represent the mean and 95% confidence interval. Points represent nine independent biological replicates (n = 9). To confirm LPS-induced NO production, control + LPS was compared with the unstimulated control using an exact two-tailed Wilcoxon matched-pairs test (## *p* = 0.0039). To determine whether the treatments altered basal NO production, each −LPS treatment group was compared with the unstimulated control using a Friedman test followed by Dunn’s multiple-comparisons test (†† adjusted *p* < 0.01 for *L. meyenii* at 0.5 and 1 mg/mL). To evaluate inhibition of LPS-induced NO production, each treatment + LPS group was compared with control + LPS using a separate Friedman–Dunn analysis (dexamethasone: adjusted *p* = 0.1610, not significant, ** adjusted *p* < 0.01 for *L. meyenii* 0.5 mg/mL and *B. gasipaes* 1 mg/mL; *** adjusted *p* < 0.001 for *L. meyenii* 1 mg/mL).

The anti-inflammatory activity of *L. meyenii* and *B. gasipaes* extracts was evaluated in LPS-stimulated RAW264.7 macrophages by measuring nitric oxide (NO) production (Figure 5a). Both extracts were evaluated at concentrations of 0.5 and 1 mg/mL, concentrations selected based on the preliminary MTT assay, which showed that they did not compromise cell proliferation (Figure S3).

Under basal conditions, NO production remained low in all non-stimulated groups, with values ranging from 0.56 to 1.28 µM. The untreated control (only medium) presented a mean NO concentration of 0.59 µM, and although basal NO production remained low, *L. meyenii* produced a slight but statistically significant increase at both concentrations compared with the untreated control. Following LPS stimulation, NO production increased markedly in the control + LPS group, reaching 11.99 µM. *L. meyenii* showed the greatest reduction in NO levels, reaching 6.86 µM at 0.5 mg/mL and 4.95 µM at 1 mg/mL, corresponding to significant reductions of 42.8% and 58.7%, respectively. Dexamethasone, used as the anti-inflammatory control, presented a mean NO concentration of 8.60 µM; however, this reduction did not reach statistical significance compared with control + LPS. *B. gasipaes* produced NO concentrations of 9.38 µM at 0.5 mg/mL and 7.72 µM at 1 mg/mL; only the higher concentration produced a statistically significant reduction (35.6%) relative to control + LPS. Crystal violet adhesion assay performed at the end confirmed that all experimental conditions maintained macrophage viability above 80% (Figure 5b), supporting that the significant decrease in NO production observed for *L. meyenii* at 1 mg/mL was not associated with reduced cell viability.

#### 2.5.2. Expression of Inflammation-Related Genes in THP-1 Cells

**Figure 6:**
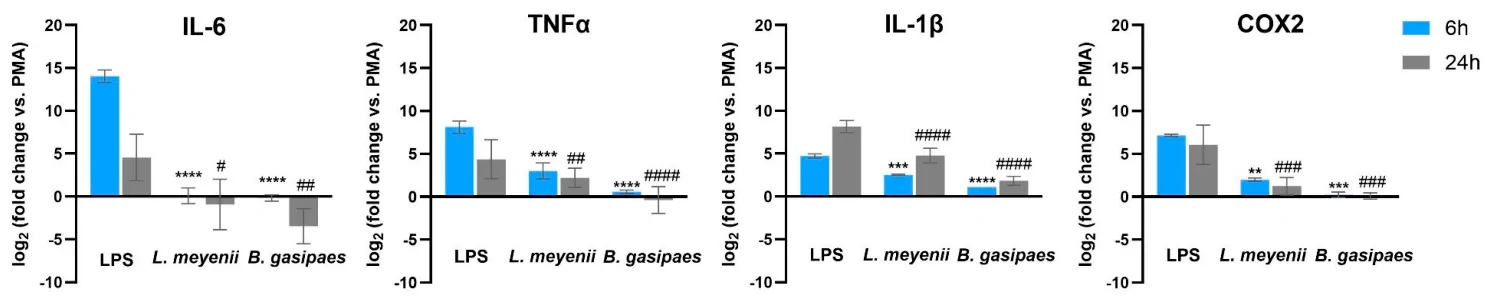
Relative expression of inflammation-related genes in THP-1 cells. PMA-differentiated THP-1 cells were treated with LPS, *L. meyenii*, or *B. gasipaes* extracts for 6 and 24 h. Gene expression is shown as log_2_(fold change) relative to the PMA control, where 0 indicates no change. Data are presented as the mean ± SD from three independent biological experiments per condition. Dunnett’s multiple-comparisons test compared each extract with the corresponding LPS condition within each time point. Significance levels were: #, *p* < 0.05; ** or ##, *p* < 0.01; *** or ###, *p* < 0.001; and **** or ####, *p* < 0.0001. Asterisks and hash symbols denote comparisons at 6 and 24 h, respectively.

The expression of inflammation-related genes was evaluated in PMA-differentiated THP-1 cells after 6 and 24 h of exposure to *L. meyenii* and *B. gasipaes* extracts (Figure 6). Gene expression was calculated as fold change relative to the PMA-only condition, and non-induced THP-1 cells were also included to assess the effect of macrophage-like differentiation. LPS was included as a pro-inflammatory reference stimulus, consistent with its use in the NO production assay, to define the inflammatory transcriptional reference profile used for comparison with the extract-treated groups.

In the LPS-treated group, a broad pro-inflammatory transcriptional profile was observed. *IL-6* was the most strongly induced early-response gene at 6 h, followed by a lower but still evident induction of *TNFα*. In contrast, *IL-1β* displayed a delayed pattern, with higher expression at 24 h, whereas *COX-2* showed sustained induction across both time points. In extract-treated cells, the temporal pattern observed for *IL-1β*, *TNFα*, and *COX-2* was similar to the LPS reference profile, but expression levels were significantly lower than those observed with LPS at both time points. At 6 h, *L. meyenii* increased the expression of *IL-1β*, *TNFα*, and *COX-2* relative to the PMA-only condition to values below 10-fold, whereas *B. gasipaes* showed minimal variation, with all evaluated genes remaining close to the PMA-only condition. At 24 h, *IL-1β* increased further, reaching approximately 27-fold with *L. meyenii* and remaining below 5-fold with *B. gasipaes*. Over the same period, *TNFα* decreased in both extract-treated groups, while *COX-2* remained low, with values close to 2-fold for *L. meyenii* and close to 1-fold for *B. gasipaes*. In contrast, *L. meyenii* maintained *IL-6* expression close to the PMA-only condition at both time points, while *B. gasipaes* remained close to basal expression at 6 h and decreased below the PMA-only condition at 24 h; both responses were significantly lower than those observed with LPS.

Overall, *L. meyenii* showed selective inflammatory gene induction and *B. gasipaes* remained largely unchanged across the evaluated markers, with neither extract reproducing the broad transcriptional response observed with LPS.

### 2.6. Bioactivity-Guided Fractionation of L. meyenii

#### 2.6.1. Antitumor Screening and Selection of Active Fractions

Although both extracts were evaluated in the whole-extract MTT screening, *L. meyenii* was selected for bioactivity-guided fractionation based on its relatively better selectivity profile compared with *B. gasipaes*, rather than on absolute antiproliferative potency alone. Cytotoxicity was then assessed using the crystal violet assay in HT-29 and MDA-MB-231 cells, which showed the most favorable IC_50_ and TI values in the whole-extract assay, and in NIH3T3 cells as a non-tumoral reference (Table 7; Figure S4). Because each fraction was tested up to its maximum feasible concentration, the results were used as a bioactivity-guided screening to prioritize active fractions. Considering the C18 SPE separation principle, early aqueous fractions were expected to contain more polar or weakly retained constituents, whereas later methanol-rich fractions were expected to be enriched in less polar or more strongly retained compounds.

None of the fractions reached 50% inhibition of cell proliferation in NIH3T3 cells within the concentration range evaluated (Table 7). Similarly, fractions F1–F5 showed no measurable IC_50_ values in MDA-MB-231 cells, whereas fractions F6–F8 exhibited antiproliferative activity. In HT-29 cells, antiproliferative activity was observed across several fractions, although the magnitude of the effect varied considerably. Fractions F2–F4 were inactive within the tested concentration range, while F1 and F5–F8 reduced cell proliferation to different extents. The strongest effects were observed for fractions F7 and F8, whereas F1 showed only limited activity. Although the activity detected in F1 suggests that some polar or weakly retained constituents may also contribute to the biological response, this effect was observed near the upper limit of the tested concentration range and should therefore be interpreted cautiously and considered secondary to the clearer activity observed in the later fractions. Based on these results, fractions F5–F8 were selected for subsequent chemical characterization.

#### 2.6.2. Chemical Characterization of Bioactive Fractions

**Figure 7:**
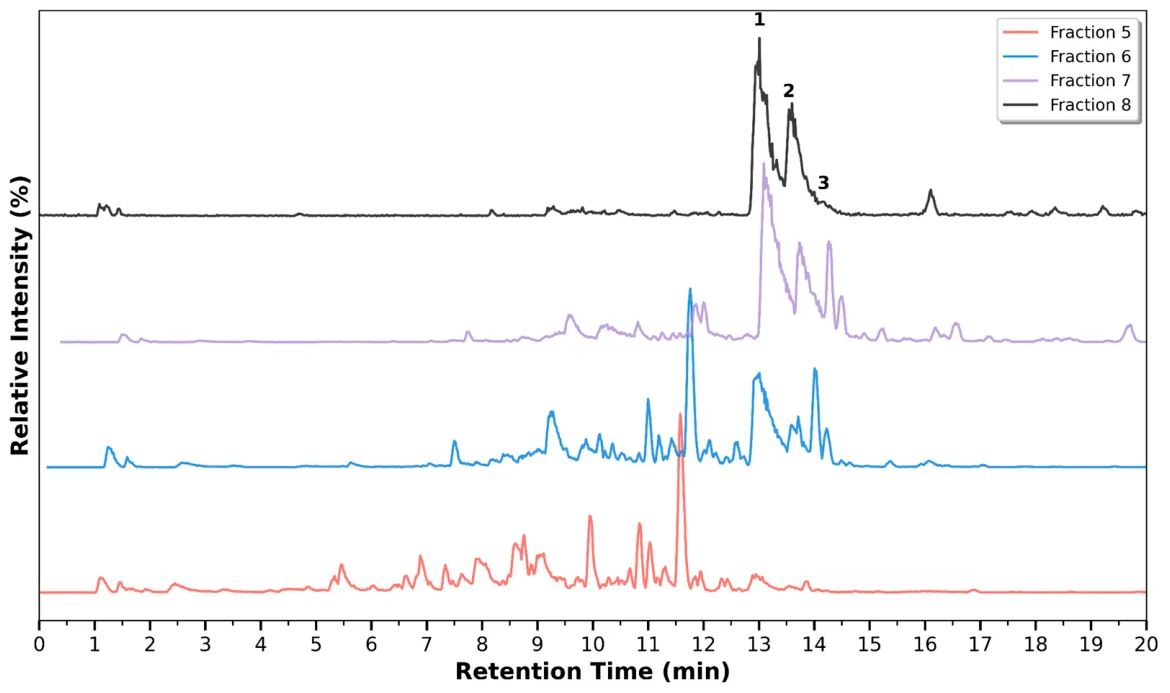
Overlaid HPLC-DAD chromatograms of fractions F5–F8 from *L. meyenii* after solid-phase extraction. Chromatograms are normalized to base peak intensity and offset vertically for clarity.

To identify candidate bioactive constituents associated with the antiproliferative effects, a bioactivity-guided fractionation approach was employed. Based on the biological screening results (Table 7), the fractions of *L meyenii* that exhibited the most promising antiproliferative profiles (F5–F8) were selected for chemical characterization using high-performance liquid chromatography coupled with electrospray ionization tandem mass spectrometry (HPLC–ESI–MS/MS) in positive ionization mode. The resulting chromatographic profiles (Figure 7) revealed that the biological response from F5 to F8 broadly paralleled a progressive chemical simplification and enrichment, although not monotonically (see below). The highly active F7 and F8 profiles were dominated by three late-eluting peaks (peaks 1–3, RT 13.01–14.17 min), indicating that the selective antiproliferative response co-purified with this hydrophobic group of metabolites.

These three peaks were putatively annotated as the imidazole alkaloids lepidilines A, B and D (Table 8). As permanently charged 1,3-dibenzyl-imidazolium salts, they were detected as intact [M]^+^ cations, and their fragmentation was governed by cleavage of the labile N-benzyl bonds. Compound ID1 (peak 1; RT 13.01 min) showed [M]^+^ at *m*/*z* 277 that, through the neutral loss of 92 Da from one N-benzyl group, generated the dominant product ion at *m*/*z* 185, together with the tropylium cation at *m*/*z* 91 (C_7_H_7_^+^), consistent with lepidiline A. Compound ID2 (peak 2; RT 13.54 min) displayed [M]^+^ at *m*/*z* 291 (+14 Da relative to ID1, indicating an additional methyl group), with a base peak at *m*/*z* 199 arising from the same 92 Da loss and the tropylium ion at *m*/*z* 91, consistent with lepidiline B. Compound ID3 (peak 3; RT 14.17 min) exhibited [M]^+^ at *m*/*z* 321, which lost 122 Da to yield *m*/*z* 199 and generated a methoxytropylium cation at *m*/*z* 121 (C_8_H_9_O^+^), diagnostic of a methoxybenzyl substituent and consistent with lepidiline D. The assignment of ID3 as lepidiline D rather than the isomeric lepidiline C is supported by its fragmentation: the loss of 122 Da to give *m*/*z* 199 indicates a trimethylimidazolium core bearing one unsubstituted benzyl group, whereas lepidiline C ([M]+ at *m*/*z* 307) would give *m*/*z* 185 through the same process. The elution order observed here (A < B < D) also reproduces that reported for these alkaloids under reversed-phase conditions [46], and lepidilines have been proposed as chemical markers reported to occur exclusively in *L. meyenii* [47].

**Figure 8:**
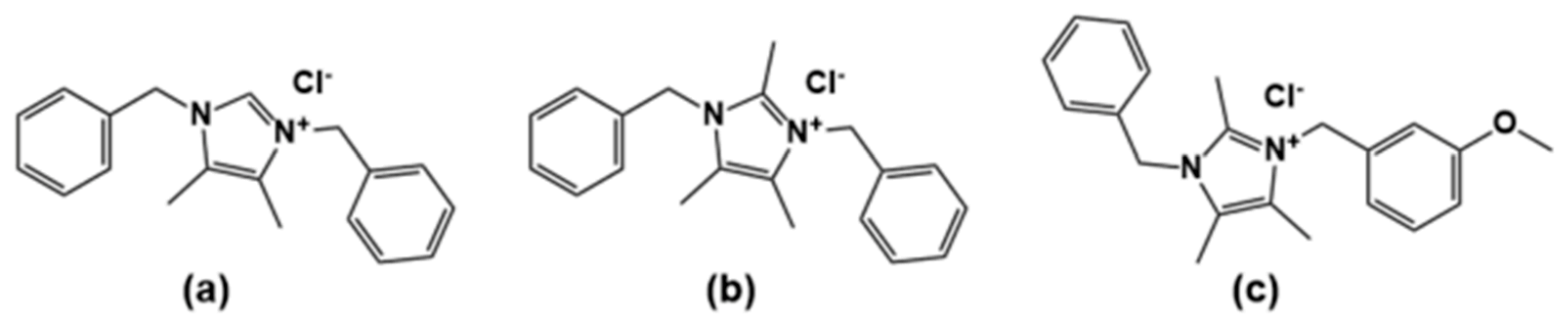
Proposed structures of (**a**) lepidiline A, (**b**) lepidiline B, and (**c**) lepidiline D, as reported previously [6,47,48,49]. The structures illustrate the annotations proposed for peaks 1–3 on the basis of their MS/MS fragmentation and relative elution order.

The three alkaloids share a common 1,3-dibenzyl-imidazolium core and differ only in ring substitution, and the diagnostic ions observed (*m*/*z* 91, 121, 185, and 199) are in agreement with the fragmentation previously described for this class of maca alkaloids [6,47,48,49]. All assignable peaks matched previously described *L. meyenii* metabolites (Figure 8).

Overall, the chemical simplicity of the most active fractions, dominated by lepidilines A, B and D, provides a coherent chemical correlate for the selective antiproliferative activity of *L. meyenii*. The criteria used for these assignments are, however, converging rather than definitive. No authentic standards were co-injected, and the linear ion trap employed operates at nominal mass, so neither retention-time matching nor accurate-mass confirmation of the molecular formulas was possible. Structural confirmation by NMR would have required isolating the compounds at preparative scale, which was beyond the scope of this work. In particular, the position of the methoxy substituent in ID3 cannot be established from MS/MS data alone. These compounds are consequently reported as putative annotations (MSI Level 2) rather than confirmed identifications.

Notably, the antiproliferative response across F5–F8 did not follow the elution gradient monotonically: in HT-29 cells, F6 showed no potency gain over F5 (IC_50_ 301.88 ± 28.33 versus 228.99 ± 28.33 µg/mL) despite eluting at a higher methanol content, whereas F7 and F8 were 3.1- and 5.8-fold more potent than F6. Because lepidiline-related signals were detected in F5–F6 as well as in F7–F8, activity did not scale with the presence of the imidazole alkaloids but paralleled the progressive loss of the co-eluting constituents that dominate the F5 and F6 profiles (Figure 7). The alkaloids may therefore be present in F5–F6 below an effective dose within the tested range, and/or co-occurring metabolites may antagonize their effect or reduce their availability to the cells. The present design cannot discriminate between these possibilities: a bioactivity-guided fractionation with a single readout per fraction establishes that activity co-purifies with a chemical class, but cannot separate an intrinsic potency difference from synergistic or antagonistic interactions, or exclude a contribution from unidentified minor components. Resolving this would require the isolated alkaloids to be evaluated alone and in defined binary combinations using fixed-ratio designs analysed by isobologram or combination-index methods. We identify this as the priority next step and therefore present lepidilines A, B and D as candidate contributors to the activity of *L. meyenii* rather than as its sole active principles.

## 3. Discussion

The biological activity of plant extracts is strongly influenced by the chemical nature of their metabolites and by the technological processes used to stabilize them. Accordingly, the present findings are discussed in relation to the contrasting composition of the two matrices and the effects of microencapsulation on their physicochemical and biological properties.

Extraction yield and metabolite recovery were determined from the total solids recovered after solvent removal [50,51,52]. The recovery of bioactive compounds is influenced by plant-related factors, including species, matrix characteristics, maturity stage and the plant part used, as well as by cultivation and processing conditions [53,54,55,56,57]. In addition, extraction parameters, including solvent type and polarity, extraction time, temperature, and agitation, may affect the efficiency and composition of the recovered metabolites, which may consequently influence the biological activity of the extracts [53,54,55,56,57,58,59]. In this context, extraction of *L. meyenii* with a 70% hydroethanolic solution favored the recovery of polar compounds, as evidenced by the presence of organic acids, mainly malic and tartaric acids; phenolic compounds, particularly 4-hydroxybenzoic acid; and alkaloids, consistent with previous studies [60,61]. Recent high-resolution metabolomic characterization of maca roots further supports the chemical diversity of this matrix, including alkaloid- and glucosinolate-related constituents, and organic acids [6,62]. In contrast, the use of 96% ethanol in *B. gasipaes* was associated with a carotenoid-dominated profile with high concentrations of β-carotene and β-cryptoxanthin, in agreement with previous studies describing this species as a relevant source of bioactive carotenoids [12,63,64]. Although organic acids and phenolic compounds have been reported in other *B. gasipaes* preparations [65,66], the targeted compounds were not detected under the analytical conditions used in this study.

The compositional differences observed between the two species also determined the selection of the encapsulating systems. Maltodextrin was employed for *L. meyenii* due to its compatibility with extracts rich in hydrophilic compounds [25,67,68], whereas the combination of maltodextrin and gum arabic was used for *B. gasipaes* because of the emulsifying capacity of gum arabic toward lipophilic compounds [44,69,70]. Microencapsulation efficiencies obtained for both formulations indicated effective retention of the selected bioactive markers, consistent with the physicochemical properties of the encapsulating materials. Maltodextrin’s high solubility and low viscosity favor low-porosity particles [25,71], while its combination with gum arabic promotes compact structures and retention of lipophilic compounds, such as carotenoids, favoring high encapsulation efficiency [69,72]. Although some extract-related components remained detectable after spray drying, this is expected in plant extract microencapsulation and does not contradict the high encapsulation efficiencies obtained; rather, it suggests that a small fraction of metabolites may remain surface-associated or accessible within the polymeric matrix [37,41,73]. Additionally, the obtained yields were within the ranges reported for similar systems, such as those described for cocoa shell polyphenols microencapsulated with gum arabic (62.2–63.7%) [74], with losses likely reflecting operational factors, such as inlet temperature and feed rate, which can favor product adhesion to the drying chamber and hinder its recovery [74,75]. Taken together, these findings indicate that both extracts were efficiently incorporated into spray-dried systems, but encapsulation efficiency, reflecting the low proportion of surface-associated markers, should be interpreted together with the physical behavior of the formulations after reconstitution and the biological activity of the reconstituted systems, rather than as a direct indicator of retained antiproliferative potency.

These matrix-specific profiles also help explain the antiproliferative responses observed for the liquid formulations. In *L. meyenii*, the antiproliferative effect may be attributed to alkaloids and phenolic compounds detected in the extract, together with other maca-associated metabolites such as macamides, metabolites previously associated with anticancer activity in different cell lines [76,77]. These compounds have been reported to affect mechanisms relevant to tumor-cell proliferation and survival, including PI3K/Akt signaling, mitochondrial function, cell-cycle progression, p53- and FoxO-associated apoptosis, and pyroptosis-related responses [78,79]. In turn, the activity observed in *B. gasipaes* may be associated with its high carotenoid content, mainly β-carotene and β-cryptoxanthin, compounds previously associated with inhibition of cell proliferation and induction of apoptosis in different tumor models [80,81,82]. Several studies indicate that β-carotene may act through mechanisms involving activation of the JNK pathway, increased caspase-3 activity, upregulation of p53, and downregulation of Bcl-2 expression [83,84]. However, the observed activity should not be attributed exclusively to β-carotene, as other co-extracted constituents, such as β-cryptoxanthin, 9-cis-violaxanthin, and terpenes, may also contribute to the biological response [85]. At the extract level, the antiproliferative activity observed here is comparable to that previously reported for *L. meyenii* extracts in Caco-2 and MDA-MB-231 cells, while evidence for *B. gasipaes* remains limited, although a recent study reported cytotoxic activity in PANC-1 cells; however, differences in extract preparation and cellular models limit direct quantitative comparison [11,86,87,88]. Importantly, although antioxidant activity has been reported for *L. meyenii* and *B. gasipaes* preparations, the low radical-scavenging activity observed under the present conditions indicates that antioxidant capacity alone does not explain the antiproliferative effects observed for either extract [60,63,89,90]. Comparative studies of maca-root extraction systems have shown that antioxidant activity varies with solvent and extraction technique, with stronger DPPH and ABTS responses reported for methanolic Ultra-Turrax extraction and aqueous shaking-assisted maceration [6,60]. The modest DPPH response observed here may also relate to the predominance of 4-hydroxybenzoic acid, the main quantified phenolic compound, which has limited activity compared with more highly hydroxylated hydroxybenzoic acids [91]. For *B. gasipaes*, greater DPPH and ABTS responses have been reported following supramolecular extraction of fruit peel [92], whereas studies using cooked and freeze-dried pulp or differently processed peel have shown that the plant portion, fruit phenotype, and drying treatment influence the recovered chemical profile [13,63].

Despite this activity, the microencapsulated extracts required higher total solid concentrations to achieve comparable antiproliferative effects, indicating that the high encapsulation efficiencies did not translate into equivalent antiproliferative potency after reconstitution. This tendency may be partly related to the physical state of the formulations during cell exposure [93]. In this regard, the aggregated structures observed by AFM in samples prepared after reconstitution showed formulation-dependent differences in size range and surface features [94,95]. These observations suggest that such structures may reflect a complex physical organization, potentially influencing compound availability and interactions with the cell monolayer and thereby contributing to the different responses of liquid and microencapsulated extracts [96,97,98]. This interpretation is consistent with reports showing that carrier properties can modify biological activity in microencapsulated systems using chitosan, where a reduction in antitumor activity against gastric and lung cancer was observed, whereas encapsulation with gum arabic enhanced such activity even at concentrations as low as 100 μg/mL [99]. Similar microencapsulated plant-extract systems have also shown formulation-dependent changes in tumor-cell inhibition and selectivity [38]. Although partial modification of thermolabile compounds during spray drying cannot be ruled out, this effect may be limited by the short exposure time associated with rapid heat and mass transfer during the process [21,96,100]. Beyond the in vitro setting, in vivo studies have demonstrated that microencapsulation can protect bioactive compounds against hostile environments, although its final effect depends on the chemical composition of the extract and the physicochemical properties of the encapsulating polymer, which determine metabolite release and bioavailability [24,101]. In the present reconstituted system, however, the reduction in biological potency following microencapsulation could reflect changes in the physical presentation and availability of active constituents, although the relative contribution of these effects versus chemical modification cannot be established from the present data. For *L. meyenii*, lepidilines provide a plausible example, as their cationic imidazolium core coupled with nonpolar substituents confers an amphiphilic character of intermediate polarity, consistent with their strong retention in reverse phase and elution in the methanol-rich F7–F8 fractions. During microencapsulation, the nonpolar domains of the lepidilines could promote hydrophobic association or self-aggregation within the highly hydrophilic maltodextrin matrix. Upon aqueous reconstitution, these interactions may contribute to incomplete redispersion of the spray-dried system, consistent with the aggregated structures observed by AFM, potentially reducing the availability of these constituents to the cells. Because different carrier systems and extract-to-carrier ratios were used for the two matrices, differences between formulations cannot be attributed independently to plant composition or formulation variables.

Beyond antiproliferative potency, the extracts also differed in their ability to affect tumor cells while maintaining lower toxicity toward non-tumor cells, a relationship commonly interpreted through the therapeutic index as a measure of efficacy–toxicity balance [102]. This phenomenon may reflect differences in the dependence of tumor and non-tumor cells on specific signaling and survival pathways, resulting in differential sensitivity to bioactive compounds [103,104]. The greater selectivity observed in *L. meyenii* may be related to the combined contribution of its bioactive metabolites, particularly polyphenols and alkaloids, which have been associated with the modulation of pathways involved in tumor-cell proliferation and survival [105,106,107,108]. Nevertheless, the presence of compounds such as steroids may partly explain why this selectivity remained moderate, since these metabolites may contribute to less selective cytotoxic effects [78]. In contrast, the lower selectivity observed in *B. gasipaes* may be related to interactions among the different metabolites present in the extract, considering that isolated β-carotene has demonstrated antitumor activity without affecting normal MCF-12A cells [84]. Likewise, microencapsulation modified selectivity in an extract-dependent manner, likely through changes in metabolite retention and availability, which may alter biological activity depending on the susceptibility of each cell line [103,109,110]. Because the TI values were calculated using murine NIH3T3 cells, with the liquid *L. meyenii* NIH3T3 IC_50_ estimated by extrapolation, confirmation of these selectivity trends in human non-tumor cells would strengthen their translational interpretation.

Within this selectivity framework, the bioactivity-guided fractionation of *L. meyenii* provided additional chemical support for its tumor-cell response, as the active fractions showed clearer antiproliferative effects in tumor cells than in NIH3T3 cells. Previous fractionation studies associated the antiproliferative activity of maca with macamide- and macaene-containing fractions [7], glucosinolate-enriched fractions [8], and, more recently, antiglioma subfractions enriched in macamides and polyunsaturated fatty acids [111]. In comparison, the present sequential C18 SPE approach localized the strongest responses in fractions F7 and F8, particularly against HT-29 cells, and extended these previous findings by characterizing their differential activity across HT-29, MDA-MB-231, and NIH3T3 cells. Consistent with the C18 SPE separation principle, the major polar metabolites quantified in *L. meyenii* would be expected to elute mainly in the early, weakly retained fractions (F1-F4), which were largely inactive. In contrast, activity increased toward the methanol-rich fractions, particularly F7 and F8, where the imidazole alkaloids lepidilines A, B, and D were putatively annotated. These compounds have been previously associated with tumor-cell-related activity in different cellular models. Although lepidiline-related signals were also detectable in the less active F5–F6 fractions, the differences in activity suggest that the biological response may depend on their relative enrichment and co-eluting constituents. Beyond differences in relative enrichment and co-eluting constituents, the biological activity of these alkaloids may be related to their structural characteristics. Lepidiline A has been reported to sensitize T-47D cells through a proposed inhibition of HSD17B1, reducing estradiol formation and limiting hormonal stimulation of tumor cells [112]. Among them, lepidiline B was one of the more active lepidilines in tumor-cell assays, while lepidilines A–D showed no evident toxicity toward normal HUVEC cells [113]. The presence of methyl groups in lepidilines B and D has been associated with increased lipophilicity, which may facilitate membrane permeation and interactions with intracellular targets involved in tumor proliferation [46]. Nevertheless, the biological activity cannot be attributed exclusively to individual alkaloids, since variation in antiproliferative response among fractions containing lepidiline-related signals may reflect differences in alkaloid abundance and/or interactions with co-eluting constituents. Thus, lepidilines A, B, and D may be considered candidate contributors to the tumor-cell response of *L. meyenii*, rather than sole determinants of the observed activity. Although supported by ESI(+) MS/MS fragmentation and relative reversed-phase elution order, the assignments of lepidilines A, B, and D remain putative because authentic standards were not co-injected and no NMR or accurate-mass confirmation was obtained. Definitive confirmation would therefore require comparison with authentic standards and/or orthogonal structural characterization by NMR and high-resolution mass spectrometry.

In addition to direct antiproliferative effects, inflammation-related responses are relevant to the biological interpretation of the extracts, since inflammatory signaling can influence tumor progression and treatment response [114]. Among the two extracts, *L. meyenii* showed the clearest macrophage-related immunomodulatory effect, reducing LPS-induced NO production in RAW264.7 cells while producing a selective transcriptional response in the separate PMA-differentiated THP-1 cells, rather than the broad profile observed with LPS. In contrast, *B. gasipaes* showed a weaker NO-reducing effect than *L. meyenii*, remaining largely unchanged across the evaluated inflammatory genes. The THP-1 panel covered early cytokine activation (*IL-6* and *TNFα*), inflammatory priming (*IL-1β*), and prostaglandin-related signaling (*COX-2*) [115]. Overall, neither extract reproduced the broad LPS-like profile, with *IL-6* notably remaining uninduced. *IL1B* showed a distinct temporal pattern with higher expression at 24 h in the LPS- and extract-treated groups, in line with some previous observations [116,117]; importantly, induction by both extracts was significantly lower than that observed with LPS. Previous reports support an immunomodulatory role for *L. meyenii*-derived preparations across different inflammatory and immune-dysregulation models, ranging from macrophage polarization and LPS-associated responses in vitro to acute hepatitis and immune alterations in vivo [5,118,119,120,121,122]. Selected flavonolignans and other constituents isolated from *L. meyenii* through aqueous or ethanol-based extraction also produced moderate inhibition of nitrite formation in LPS-stimulated macrophages [123], whereas the lignan epipinoresinol, isolated following fractionation of a crude 60% hydroethanolic extract, inhibited IL-6 production in LPS-stimulated macrophages [124]. Processing can further shape macrophage responses, as *Lactobacillus*-fermented *L. meyenii* extract produced lower NO release than the non-fermented extract [10]. In the present study, the phenolic and flavonoid-related profile of *L. meyenii* provides a relevant chemical context for its NO-reducing effect, particularly because p-coumaric acid has been reported to downregulate iNOS, COX-2, TNFα, and IL-1β in LPS-stimulated macrophages through NF-κB- and MAPK-related pathways [125]. For *B. gasipaes*, the available inflammation-related evidence is mainly associated with carotenoid-based preparations [126]. Fractions obtained from *B. gasipaes* waste have been linked to anti-inflammatory outcomes in an animal model [14], while β-carotene has been reported to suppress NO- and iNOS-related responses in LPS-stimulated macrophages [127]. The comparatively weaker NO-reducing effect and limited transcriptional response observed for *B. gasipaes* may therefore reflect differences in carotenoid content, extraction selectivity, and cellular availability.

## 4. Materials and Methods

### 4.1. Plant Material and Preparation of Liquid Extracts

*L. meyenii* (maca) was supplied as maca flour by the Andes Kinkuna company. It was harvested and processed in Cotopaxi (Ecuador) between May and June 2022. The extract was obtained by heat-assisted hydroethanolic extraction under agitation (maceration). Maca flour was mixed with 70% ethanol at a 1:10 *w*/*v* ratio and extracted at 50 °C for 30 min under constant stirring at 150 rpm. Then, the residual plant material was discarded through filtration using cloth and a Corning^®^ bottle-top vacuum filtration system equipped with a 0.2 μm cellulose nitrate membrane. The obtained extract was rotary-evaporated (IKA-Werke GmbH & Co. KG, RV8, Staufen, Germany) at 68 °C and 230 rpm to remove the alcoholic component, yielding an aqueous concentrated extract. The extract was stored in amber bottles under refrigeration at 2–8 °C, protected from light exposure.

*B. gasipaes* (chonta) was obtained from the Wholesale Market of Ambato, with origin from the city of Puyo (Ecuador) in July 2022. *B. gasipaes* fruit was cleaned thoroughly with water and soap. Fruit pulp was cut to remove the seeds and dried at 60 °C for 72 h. Then, the fruit was ground to obtain a fine powder (chonta flour). The extract was obtained by heat-assisted hydroethanolic extraction under agitation. Chonta flour was mixed with 96% ethanol at a 1:70 *w*/*v* ratio and extracted at 70 °C for 1 h under stirring at 200 rpm. The residual plant material was removed by centrifugation at 3000 rpm for 10 min. The extract obtained in the supernatant was rotary-evaporated (IKA-Werke GmbH & Co. KG, RV8, Staufen, Germany) at 68 °C and 220 rpm to remove the ethanol completely, yielding an aqueous concentrated extract. The extract was stored in amber bottles under refrigeration at 2–8 °C, protected from light exposure.

No vouchers were deposited, and species identification relied on the commercial labeling of the purchased plant materials.

The total solids content of the concentrated extracts was determined using a moisture analyzer (MLS 50-3, KERN & SOHN GmbH, Balingen, Germany). Briefly, 5 mL of each concentrated extract was placed in the analyzer and dried at 120 °C until complete removal of the liquid fraction was indicated by the equipment. A single extraction batch was prepared for each plant species, and the total solids analysis was repeated three times using aliquots from the same extract. Total solids percentage (%TS) was calculated using Equation (1):

%TS = (100-% moisture)(1)

Extraction yield was expressed as grams of total solids recovered per 100 g of dry plant material used for extraction and was calculated using Equation (2):

% EY = (Total solids recovered in the concentrated extract (g))/(Dry plant material used for extraction (g)) × 100(2)

### 4.2. Determination of Secondary Metabolites in Liquid Extracts

To determine the presence or absence of secondary metabolites, the liquid extract was combined with various reagents, as suggested by [128]. Thus, the identification of steroids and terpenoids was carried out using chloroform and sulphuric acid; phenols and tannins with 10% ferric chloride; alkaloids with 2N hydrochloric acid; flavonoids with 10% ammonia; anthraquinones with benzene and 10% ammonia; saponins with distilled water; acetogenins with 10% 3,5-dinitrobenzoic acid and potassium hydroxide. Each reaction was carried out in triplicate and the quantification in duplicate.

To quantify secondary metabolites such as organic acids, phenolic compounds and carotenoids, a 1 mL aliquot of the liquid sample was collected and filtered through a 0.45 µm PVDF filter. The quantification was carried out in triplicate. Standard solutions were prepared at a concentration of 1.0 mg/mL. Serial injections ranging from 1 to 20 µL were used to fit a linear regression model (R^2^ of 0.99). The method exhibited limits of detection (LODs) of 0.20 ppm and limits of quantification (LOQs) of 0.65 ppm. The filtered sample was subsequently injected into a high-resolution liquid chromatograph (RRLC 1200) (Agilent Technologies, Santa Clara, CA, USA) equipped with a diode array detector (DAD), operating at a mobile phase flow of 1 mL/min.

For the determination of organic acids, detection was performed at 244 nm using a YMC-Triart C18 column (120 Å pore size, 150 × 4.6 mm, 3 μm) (YMC Europe GmbH, Dinslaken, Germany). The mobile phase consisted of 0.027% sulfuric acid, and quantification was carried out using citric, malic and tartaric acid standards. Quantification was performed using calibration curves for citric, malic, and L-(+)-tartaric acids prepared from 100 mg/mL stock solutions. Serial injections ranging from 1 to 20 µL were used to fit a linear regression model (R^2^ of 0.99). The method exhibited LODs and LOQs of 0.06 and 0.17 ppm for tartaric acid, 0.13 and 0.39 ppm for malic acid, and 0.08 and 0.26 ppm for citric acid, respectively.

Phenolic compounds were analyzed at wavelengths of 280 and 370 nm using a Zorbax Eclipse Plus C18 column (4.6 × 150 mm, 5 μm) (Agilent Technologies, USA). The mobile phase comprised 0.01% formic acid in water (A) and acetonitrile (B), applying a linear gradient program: 100% A at 0 min, 95% A and 5% B at 5 min, and 50% A and 50% B at 20 min. Chromatographic peaks were identified comparing the retention time and DAD-UV–Vis absorption spectrum of each sample peak with those obtained from authentic analytical standards of rutin, quercetin, myricetin, kaempferol, quercetin glucoside, chrysin, luteolin, *p*-hydroxybenzoic acid, 3-hydroxybenzoic acid, 2-methoxybenzoic acid, 3-methoxybenzoic acid, 2,5-dihydroxybenzoic acid, ellagic acid, tannic acid, and *p*-, *m*- and *o*-coumaric acids, as well as chlorogenic acid, caffeic and ferulic acids, catechin, gallic acid, protocatechuic acid, vanillic acid, shikimic acid, syringic acid, naringenin and naringin. The major compounds were quantified using individual calibration curves at 1 mg/mL. Serial injections ranging from 1 to 20 µL were used to fit a linear regression model (R^2^ of 0.99). The method exhibited LODs and LOQs of 0.048 ppm and 0.145 ppm for caffeic acid; 0.009 ppm and 0.028 ppm for chlorogenic acid; 0.012 ppm and 0.041 ppm for ferulic acid; 0.007 ppm and 0.021 ppm for gallic acid; and 0.006 ppm and 0.014 ppm for p-hydroxybenzoic acid, respectively.

Carotenoids were determined at wavelengths of 350 and 450 nm using a YMC C30 column (3 μm particle size, 4.6 × 150 mm) (YMC Europe GmbH, Dinslaken, Germany). The mobile phase consisted of methanol (A), methyl-ter-butyl ether (B), and water (C) with a linear gradient program as follows: 95% A + 5% B + 0% C at 0 min; 95% A + 5% B + 0% C at 5 min; 95% A + 5% B + 0% C at 5 min; 89% A + 11% B + 0% C at 10 min; 75% A + 25% B + 0% C at 16 min; 40% A + 60% B + 0% C at 20 min; 15% A + 85% B + 0% C at 22 min; 90% A + 5% B + 5% C at 25 min; and 90% A + 5% B + 5% C at 28 min. Chromatographic peaks were identified using standards of α-carotene, astaxanthin, trans-β-apo-8-carotenal, lycopene, β-carotene, β-cryptoxanthin, lutein, violaxanthin, and zeaxanthin. A compound was considered identified when its retention time matched that of the corresponding standard within the predefined acceptance interval and its absorption spectrum matched the standard spectrum. The major compounds were quantified using individual calibration curves at 1 mg/mL. Serial injections ranging from 1 to 20 µL were used to fit a linear regression model (R^2^ of 0.99). Total quantified carotenoids were calculated as the sum of individual compounds. The method exhibited LODs and LOQs of 0.029 and 0.09 ppm for β-carotene, 0.00 and 0.02 ppm for lutein, and 0.003 and 0.008 ppm for zeaxanthin, respectively [129].

### 4.3. Microencapsulation of Plant Extracts

The extracts were microencapsulated using the optimal spray-drying conditions previously established in a study conducted in our laboratory. Before encapsulation, the total solids of each extract were weighed using a moisture balance (MLS 50-3, KERN & SOHN GmbH, Balingen, Germany) at 120 °C. For *L. meyenii*, a mixture was prepared with a 7:3 (*w*:*w*) ratio of total solids to maltodextrin. The mixture was agitated at 50 °C and 750 rpm for 10 min using a vertical stirrer (DLAB, OS40-PRO, China). The mixture was then spray-dried using a mini-spray dryer (B290, Büchi, Switzerland) at 150 °C inlet temperature, 80 °C outlet temperature, drying air flow 100%, and atomizer air flow 500 L/h. For *B. gasipaes*, total solids, maltodextrin, and gum arabic were combined in a 1:1:1 (*w*:*w*) ratio. The mixture was emulsified using a high-speed rotary homogenizer (BK-H6B, BIOBASE Group, Jinan, Shandong, China) at 2000 rpm for 10 min. The resulting emulsion was spray-dried using a mini-spray dryer (B290, Büchi Labortechnik AG, Flawil, Switzerland). The mixture was manually introduced through the feeding tube, and the microencapsulation process was performed under the following conditions: inlet temperature of 150 °C, outlet temperature of 90 °C, drying air flow of 100%, and atomizer air flow of 500 L/h. After spray drying, the microencapsulated products were collected and weighed using an analytical balance (Radwag Wagi Elektroniczne, N1BM1F, Radom, Poland). The resulting powders were subsequently stored in hermetically sealed Ziploc bags after complete air removal and kept in a cool, dry place protected from direct sunlight exposure. The microencapsulation procedure was independently performed twice for each formulation on separate occasions under the same processing conditions. Both batches were evaluated by FTIR to assess batch-to-batch consistency, whereas subsequent analytical and biological assays were performed using one of the prepared batches.

### 4.4. Characterization of Microencapsulated Plant Extracts

#### 4.4.1. Encapsulation Efficiency and Yield Determination

For both *L. meyenii* and *B. gasipaes*, microencapsulation yield (MY) was calculated as the percentage of weighted microencapsulated powder recovered after spray drying relative to the total mass of encapsulating polymers and extract solids initially present in the feed solution, according to Equation (3):

% MY = ((Mass of recovered microencapsulated powder)/(Mass of encapsulating polymers + extract total solids)) × 100(3)

Microencapsulation efficiency (ME) was calculated using extract-specific marker compounds and defined as the percentage of extract metabolites successfully incorporated and retained within the encapsulating matrix. The microencapsulation efficiency of *L. meyenii* was determined by quantifying polyphenols in the aqueous and ethanolic fractions, adapting the methodology of [71,130] with modifications [65]. Briefly, 1000 mg of microencapsulated sample was weighed and dissolved in 10 mL of distilled water; the same procedure was performed using absolute ethanol. Both samples were subjected to vortex mixing at maximum speed for 1 min until complete dissolution of the microencapsulated matrix in water and release of surface compounds in ethanol were achieved. The use of water enabled the disruption of the polymeric matrix and the release of the total polyphenol fraction, whereas absolute ethanol, which does not fully dissolve the maltodextrin matrix under these conditions, was used to recover the surface-accessible polyphenol fraction [74,131,132]. Subsequently, the samples were centrifuged at 3000 rpm for 5 min, and the supernatants were carefully collected without disturbing the polymeric phase. Each extract was concentrated by rotary evaporation at a maximum temperature of 30 °C to a final volume of 5 mL for both aqueous and ethanolic fractions. The obtained extracts were transferred into Eppendorf tubes, protected from light using aluminum foil, and stored at 0–10 °C until analysis. Polyphenol quantification was performed by high-performance liquid chromatography (HPLC), and microencapsulation efficiency was calculated using the polyphenol concentrations measured in the aqueous (total) and ethanolic (surface-accessible) fractions with Equation (4):

% ME (*L. meyenii*) = (([polyphenols in water] − [polyphenols in ethanol])/([polyphenols in water])) × 100 (4)

The microencapsulation efficiency percentage in *B. gasipaes* was determined by measuring β-carotene content, adapting the methodology of [44] and [133]. The total and surface β-carotene in microcapsules were quantified by solvent extraction. For total β-carotene determination, 100 mg of microencapsulated sample was weighed and dissolved in 500 µL of an ethanol:water mixture (1:1, *v*/*v*) to promote microcapsule disruption (final concentration: 200 mg/mL), followed by vortex mixing for 30 s and sonication for 5 min. Subsequently, 250 µL of hexane was added, and the mixture was centrifuged at 860× *g* for 3 min, after which the hexane phase was recovered. The extraction procedure was repeatedly performed with hexane on the remaining polar phase until complete carotenoid extraction was achieved, and all recovered organic phases were pooled into a single tube and protected from light until analysis. For the determination of surface β-carotene, 100 mg of microencapsulated sample was directly mixed with 500 µL of hexane, vortexed for 30 s, and centrifuged under the same conditions, recovering the hexane phase. Quantification was performed by spectrophotometry using a 96-well microplate at 450 nm, employing a calibration curve prepared with β-carotene standard solutions in hexane. ME was calculated from the percentage of surface β-carotene extracted with hexane relative to the total β-carotene content obtained after extraction with the ethanol:water mixture followed by hexane extraction using Equation (5):

% ME (*B. gasipaes*) = (([total β-carotene] − [surface β-carotene])/([total β-carotene])) × 100 (5)

All analyses were performed in triplicate using samples from the same batch (n = 3), and the results are expressed as mean ± SD.

#### 4.4.2. Characterization by FTIR

A drop of each liquid extract was added on the base of the infrared spectrometer (Jasco Inc., 4100 FT/IR, Easton, MD, USA), and spectra were recorded using the ATR method over a wavenumber range of 4000–500 cm^−1^, with a resolution of 4 cm^−1^ and 36 scans. A small amount of each microencapsulated extract from each of two independently prepared encapsulation batches was analyzed by FTIR under the same conditions. Readings were also performed for the encapsulating polymers, maltodextrin, and the mixture of gum arabic and maltodextrin. The obtained spectra were superimposed and analyzed.

#### 4.4.3. AFM Analysis of Reconstituted Microencapsulated Extracts

50 µL of aqueous dissolution of microencapsulated extracts (0.5 mg/mL) was deposited onto freshly cleaved mica and dried overnight under ambient conditions. A single formulation batch of each microencapsulated extract was analyzed, with three AFM images acquired from different locations of the deposited samples to assess reproducibility. A statistical analysis was performed using the length and thickness of ca. 100 and 500 observed structures for *L. meyenii* and *B. gasipaes*, respectively. AFM experiments were performed by means of a NaioAFM system (Nanosurf AG, Liestal, Switzerland), in tapping mode. The data were collected with a scan rate of 1 Hz and under ambient air conditions by using a silicon Tap190Al-G cantilever (BudgetSensors, Innovative Solutions Bulgaria Ltd., Sofia, Bulgaria), with a force constant of 48 N/m and operating at a resonant frequency of 190 kHz. Image analysis and length and thickness measurements were performed using WSxM software (Nanotec Electrónica S.L., Tres Cantos, Madrid, Spain; version 4.0) [134].

### 4.5. Antioxidant Activity of L. meyenii and B. gasipaes

The extracts of *L. meyenii* and *B. gasipaes* were evaluated using DPPH and ABTS radical scavenging assays. Stock solutions of both extracts were prepared at a concentration of 1000 mg/mL, and serial dilutions, from 500 mg/mL to 3.9 mg/mL, were used for the evaluation.

For the DPPH assay, ascorbic acid (0.05 mg/mL) was used as a positive control, and the DPPH solution was prepared at 0.1 mM in analytical-grade methanol. *L. meyenii* was prepared in milli-Q water, and *B. gasipaes* was separately prepared in water, methanol, and ethanol, due to precipitation observed when mixed with methanol. For the assay, 100 µL of each sample concentration was dispensed into a 96-well plate. Subsequently, 100 µL of the DPPH solution was added to the odd-numbered columns, while 100 µL of analytical-grade methanol was added to the even-numbered columns as blanks. The plate was then incubated at room temperature for 30 min, after which absorbance was measured at 515 nm using a Cytation 5 multimode detection system (BioTek, Winooski, VT, USA). Each concentration was analyzed in technical triplicate. Three independent DPPH experiments were performed for *L. meyenii*, and the technical replicate readings were averaged within each experiment. For *B. gasipaes*, one experiment was performed for each solvent system evaluated; these solvent conditions were not treated as independent replicates. The IC_50_ value for DPPH radical scavenging activity was calculated by fitting the percentage of scavenging activity against concentration using a four-parameter logistic sigmoidal model, with the maximum response constrained to 100% using GraphPad Prism version 11.0.1 (GraphPad Software, Boston, MA, USA).

For the ABTS assay, the ABTS radical cation was generated by mixing 7 mM ABTS with 2.45 mM ammonium persulfate (APS) and incubating the solution in the dark for 24 h at room temperature. Before use, the working solution was diluted with ultrapure water to obtain an absorbance of approximately 0.70 at 734 nm. For this assay, *B. gasipaes* extract was prepared only in ethanol, while *L. meyenii* was prepared in Milli-Q water. Trolox, used as a reference antioxidant, was prepared in PBS and serially diluted to generate a standard curve over the concentration range of 0–0.1001 mg/mL (0, 0.0031, 0.0063, 0.0125, 0.025, 0.0501, and 0.1001 mg/mL) to express the results in mg Trolox equivalents/mg extract (mg TE/mg). Ascorbic acid (50 µg/mL) was included as a positive control to verify the proper performance of the assay. Samples and ascorbic acid were diluted following the same procedure. Subsequently, 100 µL of ABTS working solution was added to each well together with the corresponding 100 µL of sample or standard. The plate was incubated in the dark for 5 min, and absorbance was measured at 734 nm using a Cytation 5 multimode detection system (BioTek, Winooski, VT, USA). For each extract, the ABTS assay was performed as a single exploratory experiment, with samples analyzed in technical triplicate. The ascorbic acid positive control was evaluated in two independent assay runs, each performed in technical triplicate.

### 4.6. Antiproliferative Activity of Liquid and Microencapsulated Extracts by MTT Assay

For the evaluation of cell proliferation, SK-MEL-103 (human melanoma, ATCC HTB-69, RRID: CVCL_6069), HT-29 (human colorectal adenocarcinoma, ATCC HTB-38, RRID: CVCL_0320), MDA-MB-231 (human breast adenocarcinoma, ATCC HTB-26, RRID: CVCL_0062), and NIH3T3 (mouse embryonic fibroblasts, ATCC CRL-1658, RRID: CVCL_0594) cell lines were cultured in DMEM (Eurobio, Les Ulis, France) supplemented with 10% fetal bovine serum (Eurobio, Les Ulis, France) and 1% penicillin–streptomycin (Thermo Fisher Scientific, Gibco, Miami, FL, USA) at 37 °C in a humidified atmosphere containing 5% CO_2_.

Cells were seeded in 96-well plates (Corning) at a density of 1.0 × 10^4^ cells per well. Liquid extracts of *L. meyenii* and *B. gasipaes* were evaluated at serial concentrations ranging from 5.00 to 0.08 mg of total solids/mL. Microencapsulated products were suspended in Milli-Q water at 143 mg/mL for *L. meyenii* and 300 mg/mL for *B. gasipaes*, corresponding to the highest stock concentrations that allowed adequate dispersion of each formulation, and subsequently diluted in culture medium to obtain final concentrations ranging from 71.50 to 0.56 mg microencapsulated product/mL. To evaluate the effect of the encapsulating polymers, maltodextrin and maltodextrin–gum arabic formulations were prepared at 500 mg/mL in Milli-Q water and tested at concentrations ranging from 50.00 to 0.79 mg/mL. Cisplatin (CDDP; Sigma-Aldrich, St. Louis, MO, USA) was used as a positive control and prepared from a 1 mg/mL stock solution in Milli-Q water and evaluated at concentrations ranging from 10.00 to 0.16 μg/mL. Each concentration for all treatments, including liquid extracts, microencapsulated extracts, encapsulating-polymer controls, and cisplatin, was tested in four technical replicates in four independent experiments. Untreated cells cultured in medium alone were used as the negative control. After 72 h of incubation, 10 μL of MTT solution (5 mg/mL) was added to each well and incubated for 1 h under standard culture conditions. The medium was then removed, and the resulting formazan crystals were dissolved in 50 μL of 100% DMSO. Absorbance was measured at 570 nm using a Cytation 5 Multi-Mode Detection System (BioTek, Winooski, VT, USA). Dose–response curves were generated using GraphPad Prism 10.2 software (GraphPad Software, Inc., La Jolla, CA, USA), considering untreated cells as the 100% proliferation control and fitting the data by nonlinear regression using a four-parameter logistic model with variable slope. IC_50_ values were defined as the concentrations corresponding to 50% inhibition of cell proliferation. For microencapsulated samples, IC_50_ values were subsequently expressed as the corresponding total solids contained in the spray-dried powder of the respective *L. meyenii* and *B. gasipaes* formulations (Tables S3, S4 and S6). When 50% inhibition was not reached within the experimentally tested concentration range, the IC_50_ was estimated by extrapolation from the fitted four-parameter logistic model. For each cell line, IC_50_ values obtained for the liquid extract and its corresponding microencapsulated formulation were compared using unpaired two-tailed Welch’s *t*-tests, and *p* values were adjusted for multiple comparisons within each plant species using the Holm–Šídák method. Therapeutic index (TI) values were determined by dividing the IC_50_ obtained in NIH3T3 cells by the corresponding IC_50_ obtained in each tumor cell line.

### 4.7. Bioactivity-Guided Fractionation and Characterization of L. meyenii Fractions

#### 4.7.1. Fractionation of *L. meyenii*

The fractionation of the aqueous extract of *L. meyenii* was performed using individual Sep-Pak C18 solid-phase extraction (SPE) columns (Waters, USA) connected to a 20-port vacuum extraction manifold assembly (Agilent Technologies, USA) equipped with a rack for 16 × 100 mm collection tubes. Each SPE column was conditioned with 10 mL of methanol, followed by 10 mL of a methanol–water solution (10:90, *v*/*v*). Subsequently, 2 mL of the aqueous extract (200 mg/mL) was loaded onto each column and allowed to pass completely through the adsorbent bed. The column was then eluted sequentially with 2 mL of methanol–water solutions at increasing proportions (*v*/*v*): 10:90, 20:80, 30:70, 40:60, 50:50, 60:40, 70:30, and 80:20. The eluates collected at each step were lyophilized, and the resulting dry extracts were resuspended in a methanol–water solution (20:80, *v*/*v*) for further analysis. The same *L. meyenii* extract batch was fractionated three times under identical chromatographic conditions, with consistent chromatographic profiles obtained across runs.

#### 4.7.2. Crystal Violet Assay for Identification of Active Fractions

Cell viability was assessed by a crystal violet adhesion assay in 96-well plates using MDA-MB-231 (human breast adenocarcinoma, RRID: CVCL_0062), HT-29 (human colorectal adenocarcinoma, ATCC: HTB-38, RRID: CVCL_0320), and NIH3T3 (mouse NIH/Swiss embryo fibroblasts, ATCC: CRL-1658, RRID: CVCL_0594). Cells were seeded at 1.5 × 10^4^ cells/well and incubated at 35 °C with 5% CO_2_. After 24 h, they were treated for 72 h with serial dilutions of the *L. meyenii* fractions, prepared from maximum concentrations of 1–2 mg/mL (Fraction 1), 1 mg/mL (Fraction 2), 0.5 mg/mL (Fractions 3 and 4), 0.250–1 mg/mL (Fractions 5 and 6), 0.1–0.4 mg/mL (Fraction 7), and 0.1 mg/mL (Fraction 8). Following treatment, cells were fixed with 4% paraformaldehyde for 20 min, stained with 0.5% crystal violet for 30 min, washed, and air-dried. Retained dye was quantified at 570 nm using a Cytation 5 multimode detection system (BioTek, Winooski, VT, USA), and untreated cells were used as the viability control. Dose–response curves were generated and IC_50_ values were calculated as described in Section 4.6. At least three independent biological experiments were performed for each fraction and cell line.

#### 4.7.3. HPLC-DAD-MS/MS Analysis of Active Fractions

Each fraction obtained from the *L. meyenii* extract was subjected to chromatographic and mass spectrometric analysis using a Vanquish UHPLC system (Thermo Fisher Scientific, Waltham, MA, USA) equipped with a dual pump and a diode array detector (DAD), coupled to an LTQ-XL mass spectrometer (Thermo Fisher Scientific, Waltham, MA, USA). Instrument control and data acquisition were performed through the Xcalibur software platform. The separation was carried out on a ZORBAX Eclipse Plus C18 analytical column (4.6 × 150 mm, 5 µm; Agilent Technologies, Santa Clara, CA, USA) maintained at 35 °C. The mobile phase was composed of water containing 0.1% formic acid (solvent A) and acetonitrile (solvent B). The gradient program started with 5% B for 0–5 min, and then increased progressively to 20% B (5–10 min), 35% B (10–20 min), 55% B (20–30 min), 70% B (30–35 min), and 90% B (35–57 min). The gradient was subsequently returned to 5% B between 57 and 60 min to re-establish the initial conditions. The flow rate was maintained at 0.2 mL/min, and the injection volume was set to 50 µL. The mass spectrometer operated in positive electrospray ionization (ESI) mode, scanning over an *m*/*z* range of 50–2000. The source parameters were configured as follows: capillary temperature 275 °C, spray voltage 4.5 kV, capillary voltage 18 V, and tube lens voltage 70 V. Metabolite annotation was performed in the fractions showing the highest activity in the crystal violet assay by matching their MS and MS/MS data with those reported in the literature and in specialized databases. Assignments were based on precursor-ion nominal *m*/*z*, diagnostic MS/MS product ions and neutral losses, and comparison with previously reported fragmentation patterns and relative reversed-phase elution order. Diode-array detection was used for peak monitoring and purity assessment only, and not as an identification criterion. No authentic standards were co-injected. Metabolites are therefore reported as putative annotations corresponding to Level 2 of the Metabolomics Standards Initiative, and not as confirmed identifications.

### 4.8. Anti-Inflammatory Activity in RAW264.7 Murine Macrophages

To evaluate the anti-inflammatory activity of *L. meyenii* and *B. gasipaes* extracts, RAW264.7 murine macrophages (ATCC No. TIB-71, RRID: CVCL_0493) were used. Cells were cultured at 37 °C in a humidified incubator with 5% CO_2_ using Dulbecco’s Modified Eagle Medium supplemented with 4.5 g/L glucose and L-glutamine (DMEM; Corning, Manassas, VA, USA), containing 10% fetal bovine serum (FBS; Eurobio, Les Ulis, France) and 1% penicillin–streptomycin (Thermo Fisher Scientific, Gibco, Miami, FL, USA). For routine passaging and subculturing, a medium mixture consisting of 30% conditioned medium and 70% fresh medium was used.

For the anti-inflammatory assay, cells were seeded in 24-well plates at a density of 3 × 10^5^ cells per well and incubated for 18–24 h under the conditions described above. Subsequently, the culture medium was replaced with FBS-free DMEM containing the corresponding treatments: DMEM alone as the negative control, dexamethasone (DEX; Sigma-Aldrich, St. Louis, MO, USA) at 2 μg/mL as a positive anti-inflammatory control, and *L. meyenii* and *B. gasipaes* extracts at two concentrations (0.5 mg/mL and 1 mg/mL). All treatments were incubated for 4 h, after which lipopolysaccharide (LPS; InvivoGen, San Diego, CA, USA) was added at a final concentration of 1 μg/mL to induce nitric oxide (NO) production in the LPS-stimulated conditions. Corresponding unstimulated conditions were maintained without LPS. Cells were then incubated for an additional 20 h. All stock solutions were prepared in DMEM.

NO production was indirectly estimated from nitrite accumulation using the Griess assay. After incubation, 50 μL of supernatant from each treatment was collected and transferred to a 96-well plate. After the addition of 50 μL of Griess reagent (Sigma-Aldrich, G4410; 50 mg/mL) to each well, the reaction mixture reached a final volume of 100 μL. DMEM alone was used as the blank, and a nitric oxide standard curve (Promega, Madison, WI, USA; G296A) ranging from 0.78 μM to 100 μM was included in the assay. Samples were incubated for 10 min at ambient temperature in the absence of light. Absorbance was subsequently determined at 540 nm using a Cytation 5 multimode reader (BioTek, Winooski, VT, USA). NO concentrations were estimated by interpolation from the standard curve after correction for the DMEM blank. Cell viability was evaluated in parallel using the crystal violet adhesion staining method described in Section 4.7.2. In addition, the MTT assay described in Section 4.6 was performed to determine the IC_50_ values of the extracts in RAW264.7 cells.

Nine independent biological replicates were performed (n = 9). Each biological replicate included all experimental conditions, with two technical replicates per condition. Technical replicate values were averaged within each biological replicate before statistical analysis. To confirm that LPS induced NO production, the unstimulated control was compared with control + LPS using a two-tailed exact Wilcoxon matched-pairs signed-rank test. To determine whether the treatments altered basal NO production, the −LPS conditions were analyzed using a Friedman test followed by Dunn’s multiple-comparisons test, with each treatment compared against the unstimulated control. To evaluate the inhibition of LPS-induced NO production, the +LPS conditions were analyzed using a separate Friedman test followed by Dunn’s multiple-comparisons test, with each treatment + LPS group compared against control + LPS. Dunn’s *p*-values were adjusted for multiple comparisons, and *p* < 0.05 was considered statistically significant. Analyses were performed using GraphPad Prism version 11.0.1 (GraphPad Software, Boston, MA, USA). Complete test statistics and adjusted *p*-values are provided in Tables S9–S11.

### 4.9. Expression of Inflammation-Related Genes

To evaluate the effect of *L. meyenii* and *B. gasipaes* extracts on the expression of inflammation-related genes, THP-1 human monocytic cells (ATCC: TIB-202, RRID: CVCL_0006) were used. Cells were cultured at 37 °C in a humidified incubator with 5% CO_2_ using RPMI-1640 medium (Corning, Manassas, VA, USA) supplemented with 10% fetal bovine serum (FBS; Eurobio, Les Ulis, France) and 1% penicillin–streptomycin (Thermo Fisher Scientific, Miami, FL, USA).

For differentiation, THP-1 cells were seeded in 6-well culture plates at a density of 3 × 10^6^ cells per well and treated with phorbol 12-myristate 13-acetate (PMA; Sigma-Aldrich, Burlington, VT, USA) at a final concentration of 10 ng/mL for 24 h. After differentiation, PMA-containing medium was removed, and cells were incubated in fresh medium for 24 h before treatment. Differentiated THP-1 cells were then exposed to the corresponding treatments, including untreated control in RPMI medium only, LPS-stimulated control at 1 µg/mL, *L. meyenii* extract at 4 mg/mL and *B. gasipaes* extract at 0.1 mg/mL. All treatments were prepared in culture medium. Cells were collected at 6 h and 24 h using trypsin, centrifuged at 2000× *g* for 8 min, and washed with sterile PBS, and the cell pellets were kept at −20 °C until processing. Total RNA was extracted using the PureLink RNA Mini Kit with on-column DNase treatment (Invitrogen, Thermo Fisher Scientific), following the manufacturer’s instructions. RNA was eluted with DNase/RNase-free water in a final volume of 100 µL. RNA concentration, purity, and integrity were determined by spectrophotometric analysis using the A260/A280 ratio and by electrophoresis in a 1% agarose gel. RNA samples were stored at −80 °C.

Complementary DNA (cDNA) was synthesized from 2 µg of total RNA using the High-Capacity cDNA Reverse Transcription Kit (Applied Biosystems, Invitrogen), according to the manufacturer’s protocol. cDNA synthesis was verified by electrophoresis in a 1% agarose gel and stored at −80 °C. Primers were designed using NCBI Primer-BLAST (Primer3, version 2.5.0) against human transcript sequences and checked for target specificity. Annealing conditions were optimized by gradient PCR using genomic DNA and cDNA templates (60 °C for *IL-6*, *IL-1β*, and *GAPDH*; 56 °C for *TNF-α* and *COX-2*). Quantitative real-time PCR was performed using PowerUp SYBR Green Master Mix (Applied Biosystems, Invitrogen) in a QuantStudio 3 Real-Time PCR System (Applied Biosystems). Each reaction contained 10 ng/µL of cDNA, 1 µM of gene-specific primers, 5 µL of SYBR Green Master Mix, and nuclease-free water in a final volume of 10 µL. Reactions were performed in optical qPCR plates compatible with the QuantStudio 3 system (Applied Biosystems, Invitrogen) using the standard cycling protocol: 50 °C for 2 min and 95 °C for 2 min, followed by 40 cycles of 95 °C for 15 s and 60 °C for 1 min, followed by melt-curve analysis. No-template and no-reverse-transcription controls were included to monitor nonspecific amplification and genomic DNA contamination. Primer specificity was verified by a single melting-curve peak in each experiment and by agarose-gel electrophoresis of representative qPCR products showing the expected amplicon size. The inflammation-related genes analyzed and primers used are detailed in Table 9. *GAPDH* was used as the reference gene. Relative gene expression was calculated using the 2^−^ΔΔCt method. The results were obtained from three independent biological experiments (n = 3), each including all treatment conditions and both time points, with each qPCR reaction performed in a technical triplicate. Technical triplicates were averaged within each biological experiment before statistical analysis. Log_2_-transformed *IL6*, *IL1B*, *TNF-α*, and *COX2* expression values were analysed separately using two-way mixed-effects models fitted by restricted maximum likelihood (REML), with treatment and time as fixed effects and independent experiment as the matching factor. Dunnett’s multiple-comparisons test was used to compare each extract with LPS within each time point. Analyses were performed using GraphPad Prism version 11.0.1 (GraphPad Software, Boston, MA, USA), with adjusted *p* < 0.05 considered statistically significant.

## 5. Conclusions

This study highlights the value of integrating chemical characterization, formulation analysis, and biological evaluation when assessing edible plant extracts with antitumor-relevant potential. Among the two matrices, the liquid extract of *L. meyenii* combined tumor-cell inhibition, moderate selectivity, and an inflammation-modulating response that did not reproduce a broad LPS-like inflammatory pattern. In contrast, *B. gasipaes* showed antiproliferative activity but a less consistent selectivity profile under the evaluated conditions. These comparisons should be interpreted within the specific extraction and formulation conditions used for each plant matrix. Microencapsulation generated extract-loaded systems with high encapsulation efficiencies; however, the biological activity observed after reconstitution depended on the plant matrix and cell line evaluated. This indicates that encapsulation efficiency should be interpreted together with post-reconstitution biological activity and highlights the need to refine formulation conditions aimed at preserving activity. Bioactivity-guided fractionation of *L. meyenii* further linked the observed tumor-cell response to less polar fractions enriched in signals putatively annotated as lepidiline-type imidazole alkaloids, including lepidilines A, B, and D. Because these assignments were based on MS/MS fragmentation and relative elution order, without co-injection of authentic standards or NMR confirmation, they should be regarded as putative annotations requiring verification in future work. Across both the formulation and fractionation experiments, matrix chemistry and spray-dried formulation jointly shaped the in vitro behavior of the extracts. Taken together, these results identify the liquid extract of *L. meyenii* as having the most favorable antitumor-relevant profile under the specific in vitro conditions evaluated in this study based on its balance of antiproliferative activity, selectivity, and inflammation-related effects. Future work should validate the activity of isolated or enriched constituents in broader tumor and non-tumor models, and monitor liquid and spray-dried extracts during storage to explore whether microencapsulation preserves bioactive markers and biological activity over time.

## Figures and Tables

**Table 1:** Extraction conditions and recovery parameters of the liquid plant extracts.

Plant Species	Solvent	Extraction Time (min)	Ratio (g/mL)	Total Solids (mg/mL)	Yield (g/100 g)
*L. meyenii*	70% Ethanol	30	1:10	421.4 ± 2.7	39.0
*B. gasipaes*	96% Ethanol	60	1:70	721.8 ± 1.3	14.4

Time of extraction: duration of the extraction process. Ratio: mass of dry plant material (g) per volume of solvent used (mL). Total solids: concentration of extracted compounds in the liquid extract, values are expressed as mean ± SD of three technical measurements (n = 3) from a single extraction batch. Yield: mass of total solids obtained in the concentrated extract (g) divided by the mass of dry plant material used (g).

**Table 2:** Phytochemical screening of *L. meyenii* and *B. gasipaes* extracts using different solvent systems.

Metabolites	*L. meyenii*			*B. gasipaes*		
	Methanol	Ethanol	Water	Methanol	Ethanol	Water
Steroids	−	−	+	−	+	+
Terpenoids	−	−	−	−	+	+
Phenols	+	+	−	+	+	+
Tannins	+	−	−	+	+	−
Alkaloids	−	−	+	+	+	−
Flavonoids	+	+	−	−	−	−
Anthraquinones	−	−	−	−	−	−
Saponins	−	−	−	−	−	−

+, positive color reaction; −, negative color reaction. The liquid extracts were resuspended in each of the three solvents, and each metabolite type was determined in duplicate for each extract–solvent combination. The liquid extracts were resuspended in each of the three solvents, and each qualitative reaction was performed in duplicate (n = 2) for each extract–solvent combination using aliquots from a single extraction batch for each species.

**Table 3:** Average concentrations of bioactive compounds in *L. meyenii* (mean ± SD of three technical replicate measurements from a single extraction batch).

Metabolites	Subclassification	Concentration (mg/100 mL)
Organic acids	Citric acid	537.0 ± 3.8
	Malic acid	3016.1 ± 25.3
	Tartaric acid	1650.1 ± 15.8
	Total organic acids	5203.2 ± 37.4
Phenolics	Gallic acid	34.8 ± 0.2
	4-Hydroxybenzoic acid	406.0 ± 0.9
	Syringic acid	51.6 ± 1.2
	p-coumaric acid	61.3 ± 0.9
	Naringenin	67.7 ± 0.6
	Total phenolics	621.5 ± 1.7
Alkaloids	Total alkaloids	9.067± 0.027

**Table 4:** Average concentrations of bioactive compounds in *B. gasipaes* (mean ± SD of three technical replicate measurements from a single extraction batch).

Metabolites	Subclassification	Concentration (mg/100 mL)
Carotenoids	9-cis violaxanthin	23.2 ± 2.5
	β-cryptoxanthin	39.5 ± 3.3
	β-carotene	1261.9 ± 11.8
	Total carotenoids	1324.7 ± 12.4

**Table 5:** Encapsulation efficiency, yield, and structural conformation of microencapsulated extracts.

Extract	Structural Conformation	Encapsulating Matrix	Encapsulation Efficiency (%)	Yield (%)
*L. meyenii*	Microspheres	Maltodextrin	97.71 ± 0.05	63.91
*B. gasipaes*	Microcapsules	Maltodextrin–gum arabic	97.03 ± 0.47	66.94

Encapsulation efficiency was calculated from the relationship between surface-associated and total bioactive compounds in the microencapsulated material, and values are expressed as mean ± SD of three technical replicates (n = 3) obtained from a single microencapsulated batch. Yield was calculated from the mass of dried microencapsulated material recovered after spray drying relative to the initial mass of encapsulating matrix and extract solids.

**Table 6:** Antioxidant activity of *L. meyenii* and *B. gasipaes* extracts determined by DPPH and ABTS assays.

Sample	DPPH IC_50_ (mg/mL)	ABTS Activity (mg TE/mg Extract)
Ascorbic acid	0.0039 ± 3.99 × 10^−4^	1.29 ± 2.12 × 10^−2^
*L. meyenii*	524.55 ± 0.89 × 10^2 a^	2.61 × 10^−5 b^
*B. gasipaes*	ND	1.30 × 10^−5 b^

DPPH values are mean ± SEM from three independent experiments (n = 3) and the ABTS value for ascorbic acid is mean ± SD from two independent assay runs (n = 2). ^a^ Model-estimated IC_50_ above the maximum tested concentration. ^b^ Single exploratory assay. ND, 50% inhibition was not reached; TE, Trolox equivalents. mg TE/mg sample represents the antioxidant activity equivalent to milligrams of Trolox per milligram of tested sample. Detailed replication and assay conditions are provided in Section 4.5.

**Table 7:** Bioactivity profile of *L. meyenii* fractions (F1–F8) evaluated in NIH3T3, MDA-MB-231, and HT-29 cells.

Fractions of *L. meyenii*	NIH3T3	MDA-MB-231	HT-29
	IC_50_ Values (mg/mL)		
F1	--	--	1.008 ± 0.136 ^a^
F2	--	--	--
F3	--	--	--
F4	--	--	--
F5	--	--	0.229 ± 0.032
F6	--	0.437 ± 0.024 ^a^	0.302 ± 0.028
F7	--	0.194 ± 0.013 ^a^	0.096 ± 0.007
F8	--	0.201 ± 0.017 ^a^	0.052 ± 0.006

Values are presented as mean ± SEM from at least three independent biological experiments. --Indicates that IC_50_ was not reached at the maximum concentration tested. ^a^ Indicates a model-estimated or borderline response close to or above the maximum tested concentration.

**Table 8:** Imidazole alkaloids putatively annotated by HPLC–ESI–MS/MS in positive ionization mode from the bioactive fractions (F5–F8) of *L. meyenii*.

ID	RT (min)	*m*/*z*	MS/MS	Putative Annotation	Reference
1	13.01	277 [M]^+^	91(70) 109(10) 181(15) 185(100) 199(5)	Lepidiline A	[48]
2	13.54	291 [M]^+^	91(40) 199(100) 250(5)	Lepidiline B	[6]
3	14.17	321 [M]^+^	121(100) 199(50) 229(30) 280(5)	Lepidiline D	[49]

**Table 9:** Target genes and primer sequences (5′-3′) used for inflammation-related gene expression analysis.

Gene Name	Forward Primer	Reverse Primer
*IL-6*	CATCCTCGACGGCATCTCAG	ACCAGGCAAGTCTCCTCATTG
*IL-1β*	CCACAGACCTTCCAGGAGAATG	GTGCAGTTCAGTGATCGTACAGG
*TNF-ɑ*	CCTTCCTGATCGTGGCAG	GCTTGAGGGTTTGCTACAAC
*COX-2*	CAAGACAGATCATAAGCGAGGG	GAGTATCTTTGACTGTGGGAGG
*GAPDH*	AGAAGGCTGGGGCTCATTTG	AGGGGCCATCCACAGTCTTC

## Data Availability

The original contributions presented in this study are included in the article/Supplementary Materials. Further inquiries can be directed to the corresponding author.

## Supplementary Materials

The following supporting information can be downloaded at: https://www.mdpi.com/article/10.3390/molecules31183286/s1, Table S1: Analysis of alkaloid concentration in the *L. meyenii* extract; Table S2: Encapsulation efficiency of *L. meyenii* and *B. gasipaes* extracts; Table S3: Concentration series of microencapsulated *L. meyenii* used in the MTT assay, showing spray-dried powder, equivalent total solids, and maltodextrin concentrations (mg/mL); Table S4: Concentration series of microencapsulated *B. gasipaes* used in the MTT assay, showing spray-dried powder, equivalent total solids, and maltodextrin–gum arabic concentrations (mg/mL); Table S5: Inhibitory concentration values (IC_50_) (mg/mL) of liquid and microencapsulated extracts against tumor and non-tumor cell lines at 72 h and therapeutic index (TI) values; Table S6: Quantification of total solids and polymer content of the microencapsulated extracts at IC_50_ concentrations (mg/mL); Table S7: IC_20_ values of the encapsulating polymers; Table S8: IC_50_ values of the positive control (cisplatin); Table S9: Statistical analysis of basal NO production in unstimulated RAW264.7 macrophages: Friedman test and Dunn’s multiple comparisons versus control; Table S10: Statistical analysis of NO production in LPS-stimulated RAW264.7 macrophages: Friedman test and Dunn’s multiple comparisons versus control + LPS; Table S11: Statistical comparison of NO production between control and control + LPS: exact two-tailed Wilcoxon matched-pairs signed-rank test; Supplementary Note S1: Antioxidant activity; Supplementary Note S2: Statistical analysis of THP-1 gene expression data; Figure S1: DPPH assay of *L. meyenii* and B. gasipaes; Figure S2: ABTS radical scavenging activity and Trolox calibration curves; Figure S3: Dose–response curves of *L. meyenii* and *B. gasipaes* extracts in RAW264.7 macrophages after 72 h of exposure were determined by the MTT assay; Figure S4: Dose–response effect of *L. meyenii* fractions on cell proliferation.

## Abbreviations

The following abbreviations are used in this manuscript:

ABTS	2,2′-Azino-bis(3-ethylbenzothiazoline-6-sulfonic acid)
AFM	Atomic force microscopy
APS	Ammonium persulfate
CDDP	Cisplatin
cDNA	Complementary DNA
COX-2	Cyclooxygenase-2
DEX	Dexamethasone
DMSO	Dimethyl sulfoxide
DPPH	2,2-Diphenyl-1-picrylhydrazyl
ESI	Electrospray ionization
FBS	Fetal bovine serum
FTIR	Fourier transform infrared spectroscopy
GAPDH	Glyceraldehyde-3-phosphate dehydrogenase
HPLC	High-performance liquid chromatography
IL	Interleukin
LPS	Lipopolysaccharide
MD	Maltodextrin
MS	Mass spectrometry
MS/MS	Tandem mass spectrometry
MTT	3-(4,5-Dimethylthiazol-2-yl)-2,5-diphenyltetrazolium bromide
NO	Nitric oxide
PBS	Phosphate-buffered saline
qPCR	Quantitative polymerase chain reaction
SEM	Standard error of the mean
TNFα	Tumor necrosis factor α
DMEM	Dulbecco’s modified Eagle medium
RPMI	Roswell Park Memorial Institute medium
PMA	Phorbol 12-myristate 13-acetate
SD	Standard deviation
SDG	Sustainable Development Goal
iNOS	Inducible nitric oxide synthase
UHPLC	Ultra-high-performance liquid chromatography
UV–Vis	Ultraviolet–visible spectroscopy
