# Supplementary material for: Antitumor-Relevant Evaluation of Liquid and Microencapsulated Lepidium meyenii and Bactris gasipaes Extracts Reveals Species- and Formulation-Dependent Activity
Source: Molecules. 2026 Sep 16;31(18):3286. doi: 10.3390/molecules31183286 (PMC13609216; doi:10.3390/molecules31183286)
Supplement: Supplementary file 1 [file molecules-31-03286-s001.zip › molecules-4489614-supplementary.pdf]

Supplementary Material

Antitumor-Relevant Evaluation of Liquid and Microencapsulated *Lepidium meyenii* and *Bactris gasipaes* Extracts Reveals Species- and Formulation-Dependent Activity

Table S1. Analysis of alkaloid concentration in the *L. meyenii* extract.

Evaluated parameter	Analytical technique	Temperature (°C)	Relative humidity (%)	Laboratory	Obtained value (mg/L)
Total alkaloids in *L. meyenii* extract	Spectrophotometry based on the bromocresol blue complex	24.1	50.0	Analytical Laboratories, Guayaquil	90.67± 0.27

Note: Values are expressed as mean ± SD of three technical measurements from a single extraction.

Table S2. Encapsulation efficiency of *L. meyenii* and *B. gasipaes* extracts.

Plant species	Method	Metabolite	Total (µg/mL)	Surface (µg/mL)	Efficiency (%)
L. meyenii	HPLC	Total polyphenol	1750.00 ± 0.04	40.00 ± 0.00	97.71 ± 0.05
B. gasipaes	Spectrophotometry (450 nm)	β-carotene	23.63 ± 1.63	0.70 ± 0.16	97.03 ± 0.47

Note: Values are expressed as mean ± SD of three technical measurements from a single batch.

Table S3. Concentration series of microencapsulated *L. meyenii* used in the MTT assay, showing spray-dried powder, equivalent total solids, and maltodextrin concentrations (mg/mL).

Microencapsulated *L. meyenii*	Total solids	Maltodextrin
71.5	50.05	21.45
35.75	25.02	10.73
17.88	12.52	5.36
8.94	6.26	2.68
4.47	3.13	1.34
2.23	1.56	0.67
1.12	0.78	0.34
0	0	0

Table S4. Concentration series of microencapsulated *B. gasipaes* used in the MTT assay, showing spray-dried powder, equivalent total solids, and maltodextrin–gum arabic concentrations (mg/mL).

Microencapsulated *B. gasipaes*	Total solids	Maltodextrin–Gum arabic
71.5	23.83	47.67
35.75	11.92	23.83
17.88	5.96	11.92
8.94	2.98	5.96
4.47	1.49	2.98
2.23	0.74	1.49
1.12	0.37	0.75
0	0	0

Table S5. Inhibitory concentration values (IC_{50}) (mg/mL) of liquid and microencapsulated extracts against tumor and non-tumor cell lines at 72 h and therapeutic index (TI) values.

		SK-MEL-103		MDA-MB-231		HT29		NIH3T3
		IC_{50}	TI	IC_{50}	TI	IC_{50}	TI	IC_{50}
L. meyenii	Liquid	2.96 ± 0.09	2.28	2.82 ± 0.39	2.39	1.85 ± 0.37	3.64	6.74 ± 1.12[a]
	Microencapsulated	3.51 ± 0.18	1.53	2.84 ± 0.23	1.89	2.79 ± 0.31	1.93	5.38 ± 0.63
B. gasipaes	Liquid	0.33 ± 0.26	0.97	0.27 ± 0.12	1.19	0.63 ± 0.12	0.51	0.32 ± 0.10
	Microencapsulated	1.52 ± 0.25	1.07	1.34 ± 0.21	1.22	1.18 ± 0.20	1.39	1.64 ± 0.16

Note: TI= IC_{50} (NIH3T3)/IC_{50} (tumor cell); [a] IC_{50} estimated by extrapolation, as 50% inhibition was not reached within the tested concentration range. Values are expressed as mg of total solids/mL, mean ± SD, from four independent biological experiments (n=4).

Table S6. Quantification of total solids and polymer content of the microencapsulated extracts at IC_{50} concentrations (mg/mL).

Cell line	*L. meyenii*			*B. gasipaes*		
	IC_{50}	Total solids	Maltodextrin	IC_{50}	Total solids	Maltodextrin-Gum arabic
SK MEL 103	5.02	3.51	1.51	4.57	1.52	3.05
MDA MB 231	4.06	2.84	1.22	4.02	1.34	2.68
HT-29	3.98	2.79	1.19	3.54	1.18	2.36
NIH3T3	7.68	5.38	2.30	4.91	1.64	3.27

Table S7. IC_{20} values of the encapsulating polymers.

Cell line	Encapsulating agent (mg/mL)	
	**Maltodextrin**	**Maltodextrin–Gum arabic**
SK-MEL-103	35.4	15.06
MDA-MB-231	88[a]	13.92
HT-29	48	8.31
NIH3T3	21.45	11.07

Note: [a] IC_{20} estimated by extrapolation, as 20% inhibition was not reached within the tested concentration range.

Table S8. IC_{50} values of the positive control (cisplatin).

Cell line	IC_{50} (mg/mL)
SK-MEL-103	0.006 ± 0.00053
MDA-MB-231	0.006 ± 0.00357
HT-29	0.003 ± 0.00020
NIH3T3	0.004 ± 0.00038

Note: Values are expressed as mean (n=4).

Table S9. Statistical analysis of basal NO production in unstimulated RAW264.7 macrophages:

Comparison	Rank sum difference	Adjusted P value	Significance
Control vs DEX	-17	0.1610	ns
Control vs *L. meyenii* 0.5 mg/mL	-26.000	0.0053	††
Control vs *L. meyenii* 1 mg/mL	-29.00	0.0013	††
Control vs *B. gasipaes* 0.5 mg/mL	0	>0.9999	ns
Control vs *B. gasipaes* 1 mg/mL	3.00	>0.9999	ns

Friedman test and Dunn's multiple comparisons versus Control.

Note: Basal NO production was analyzed using a Friedman test with biological replicates treated as matched subjects. The overall analysis identified a significant difference among the six conditions (Friedman statistic = 35.35, $p < 0.0001$). Dunn's multiple-comparisons test was subsequently used to compare each −LPS treatment group with the unstimulated Control. Reported p-values were adjusted for a family of five comparisons. Nine independent biological replicates were analyzed (n = 9), with two technical replicates per condition. Technical duplicate readings were averaged within each biological replicate and were not treated as independent experimental units. †† adjusted $p < 0.01$ versus Control; ns, not significant. DEX, dexamethasone

Table S10. Statistical analysis of NO production in LPS-stimulated RAW264.7 macrophages:

Comparison (+LPS)	Rank sum difference	Adjusted P value	Significance
Control vs DEX	17	0.1610	ns
Control vs *L. meyenii* 0.5 mg/mL	27.00	0.0033	**
Control vs *L. meyenii* 1 mg/mL	32.00	0.0003	***
Control vs *B. gasipaes* 0.5 mg/mL	13.00	0.5073	ns
Control vs *B. gasipaes* 1 mg/mL	28.00	0.0021	**

Friedman test and Dunn's multiple comparisons versus Control + LPS.

Note: NO production under LPS-stimulated conditions was analyzed using a Friedman test with biological replicates treated as matched subjects. The overall analysis identified a significant difference among the six conditions (Friedman statistic = 22.65, $p = 0.0004$). Dunn's multiple-comparisons test was subsequently used to compare each treatment + LPS group with Control + LPS. Reported p-values were adjusted for a family of five comparisons. Nine independent biological replicates were analyzed ($n = 9$), with two technical replicates per condition. Technical duplicate readings were averaged within each biological replicate and were not treated as independent experimental units. ** adjusted $p < 0.01$ and *** adjusted $p < 0.001$ versus Control + LPS; ns, not significant. DEX, dexamethasone; LPS, lipopolysaccharide.

Table S11. Statistical comparison of NO production between Control and Control + LPS: exact two-

Comparison	Matched pairs	Median paired difference (µM)	Wilcoxon W	Exact two-tailed p-value	Significance
Control + LPS vs. Control	9	8.340	45.00	0.0039	##

tailed Wilcoxon matched-pairs signed-rank test.

Note: Control + LPS was compared with the corresponding unstimulated Control to confirm LPS-induced NO production. The difference was calculated as Control + LPS minus Control. The analysis included nine matched biological replicates and no tied differences. Each biological replicate contained two technical replicates per condition, which were averaged before statistical analysis and were not treated as independent experimental units. ## $p < 0.01$. LPS, lipopolysaccharide.

Supplementary Note S1. Antioxidant activity

In the DPPH assay, the aqueous extract of *L. meyenii* showed weak radical-scavenging activity, with a model-estimated IC_{50} of 524.55 mg/mL, above the maximum concentration tested, compared with 0.0039 mg/mL for ascorbic acid (Figure S1A). For *B. gasipaes*, precipitation occurred when the aqueous extract was combined with methanol. The extract was therefore evaluated separately in water, methanol, and ethanol. Under all three solvent conditions, DPPH inhibition remained below 25% at the highest concentration tested and 50% inhibition was not reached (Figure S1B).

In the ABTS assay, Trolox produced approximately 78% decolorization at 0.1001 mg/mL, whereas ascorbic acid reached approximately 50% at 0.05 mg/mL (Figure S2). Based on the Trolox calibration curve, ascorbic acid showed an activity of $1.29 \pm 2.12 \times 10^{-2}$ mg TE/mg sample. In comparison, *L. meyenii* and *B. gasipaes* showed activities of 2.61×10^{-5} and 1.30×10^{-5} mg TE/mg extract, respectively. At the highest concentration tested, ABTS decolorization reached approximately 9% for *L. meyenii* and 3% for *B. gasipaes* (Figure S2). These exploratory results indicate negligible ABTS activity for both extracts under the tested conditions.

Supplementary Note S2. Statistical analysis of THP-1 gene expression data

The mixed-effects analysis showed a significant treatment effect for all genes (all $p < 0.0001$). The time effect was significant for IL-6 ($p = 0.0007$) and IL-1β ($p = 0.0118$), but not for TNFα ($p = 0.1173$) or COX-2 ($p = 0.4155$). Treatment × time interactions were significant for IL-6 ($p = 0.0129$), TNFα ($p = 0.0246$), and IL-1β ($p = 0.0005$), but not for COX-2 ($p = 0.8009$). Compared with the corresponding LPS condition, Dunnett-adjusted *p* values at 6 and 24 h, respectively, were: IL-6, *L. meyenii* < 0.0001 and 0.0177 and *B. gasipaes* < 0.0001 and 0.0017; TNFα, *L. meyenii* < 0.0001 and 0.0033 and *B. gasipaes* < 0.0001 at both times; IL-1β, *L. meyenii* = 0.0003 and < 0.0001 and *B. gasipaes* < 0.0001 at both times; and COX-2, *L. meyenii* = 0.0041 and 0.0007 and *B. gasipaes* = 0.0006 and 0.0002.

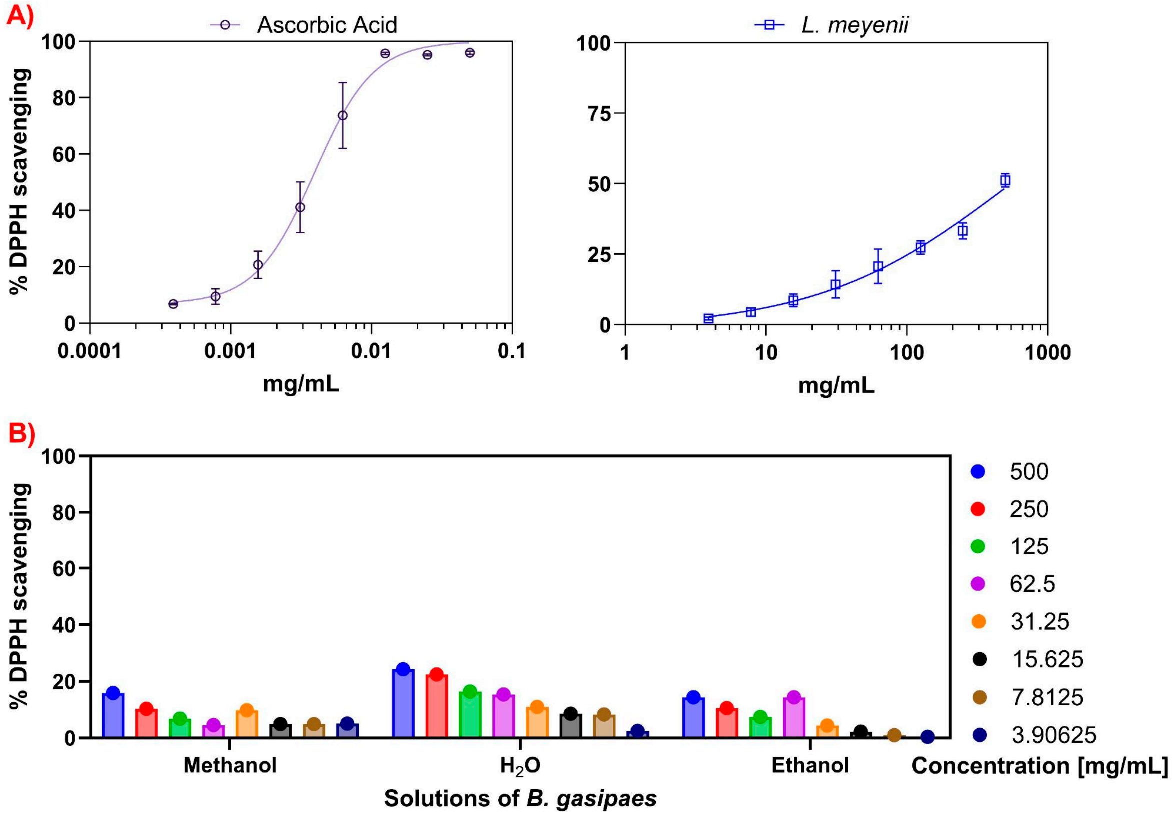

Figure S1. DPPH assay of *L. meyenii* and *B. gasipaes*. (A) Dose–response curves used for IC_{50} determination of the aqueous extract of *L. meyenii* and ascorbic acid, used as the positive control. The calculated IC_{50} values were 524.55 ± 0.89 × 10^2 mg/mL for *L. meyenii* and 0.0039 ± 3.99 × 10^{-4} mg/mL for ascorbic acid. Data are presented as mean ± SEM from three independent experiments (n = 3). (B) % DPPH scavenging activity of serial dilutions of *B. gasipaes* in different solvents.

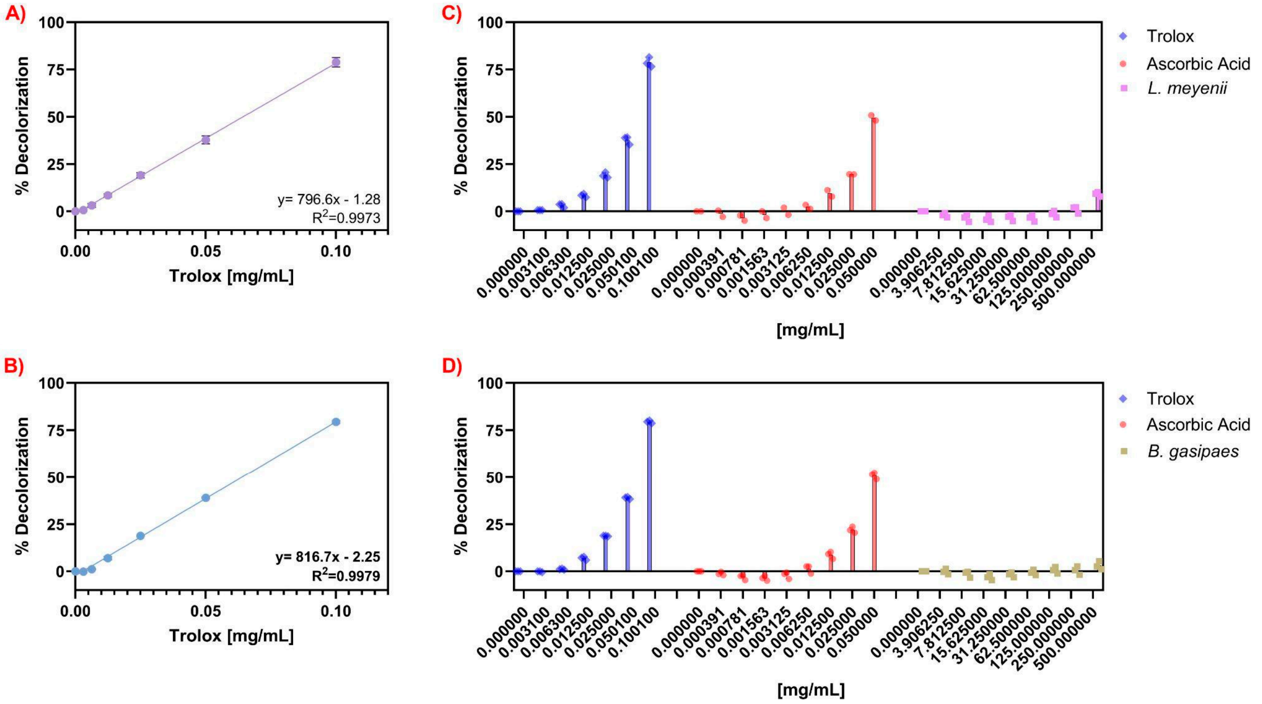

Figure S2. ABTS radical scavenging activity and Trolox calibration curves. (A–B) Calibration curves of Trolox showing the relationship between concentration (mg/mL) and percentage of decolorization (R^2 = 0.9973 and 0.9979, respectively). (C) ABTS radical scavenging activity of Trolox (blue), ascorbic acid (red), and *L. meyenii* extract (purple) across the tested concentration ranges. Trolox and ascorbic acid showed a concentration-dependent increase in decolorization, whereas *L. meyenii* exhibited minimal activity even at the highest concentrations. (D) ABTS radical scavenging activity of Trolox (blue), ascorbic acid (red), and *B. gasipaes* extract (brown) showed negligible decolorization across all tested concentrations.

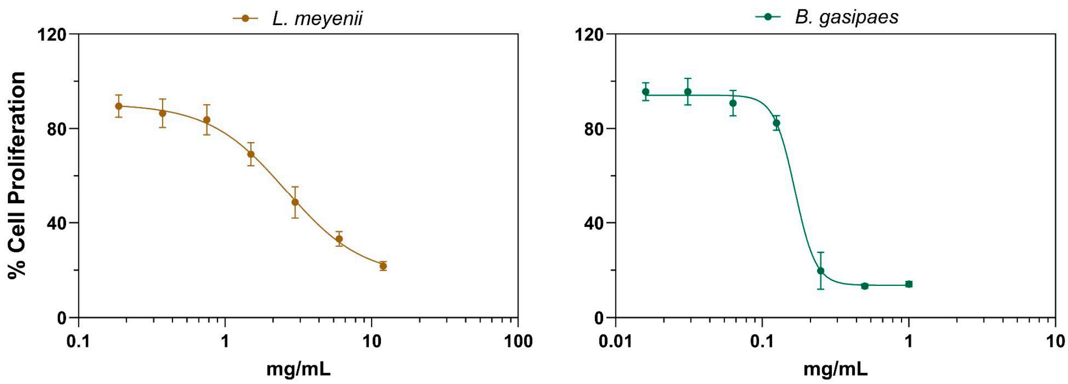

Figure S3. Dose–response curves of *L. meyenii* and *B. gasipaes* extracts in RAW264.7 macrophages after 72 h of exposure were determined by the MTT assay. IC_{50} values were obtained by nonlinear regression analysis. Error bars represent SD.

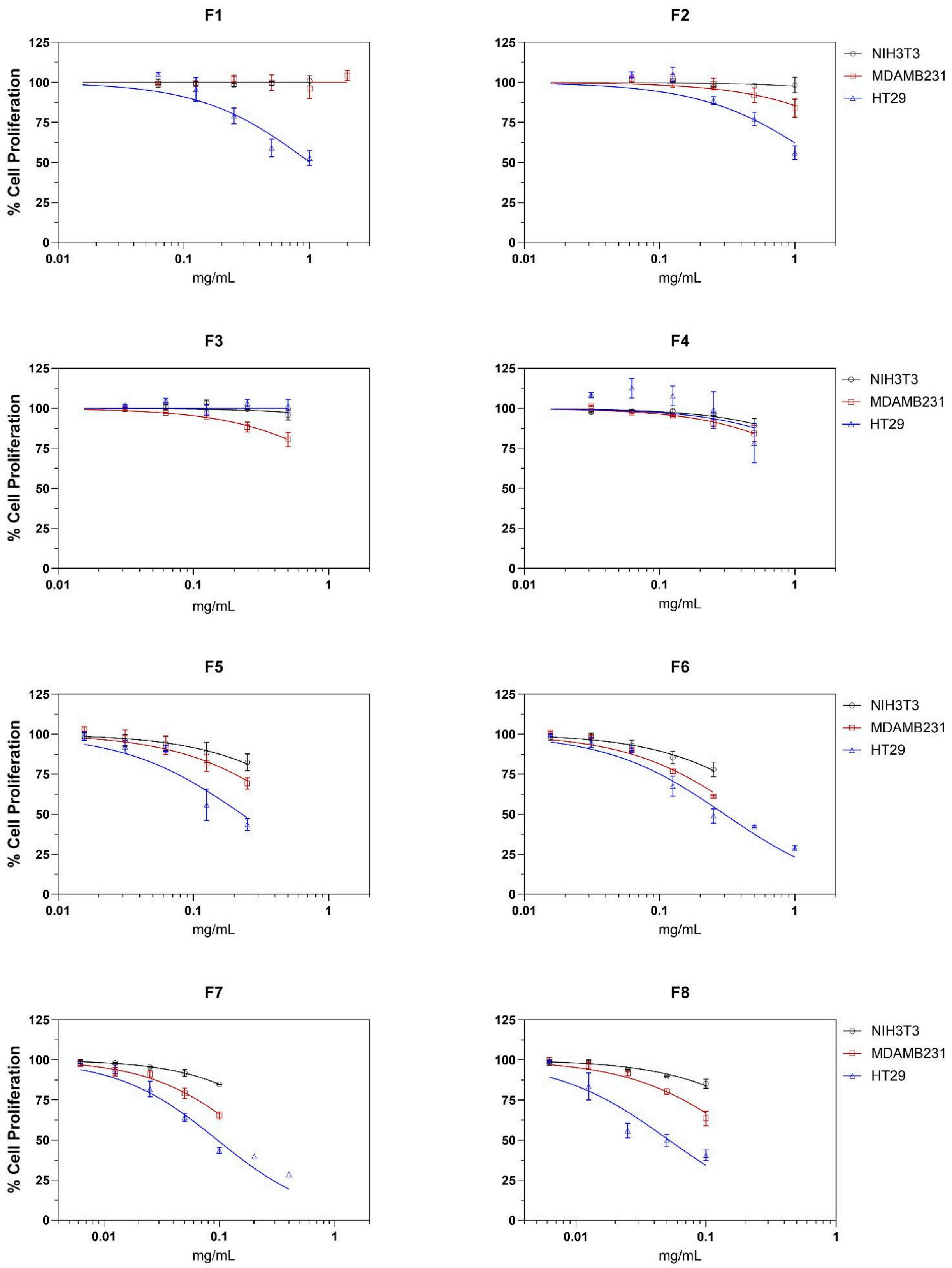

Figure S4. Dose–response effect of *L. meyenii* fractions on cell proliferation. Dose–response curves of fractions F1–F8 from *L. meyenii* evaluated in NIH3T3, MDA-MB-231, and HT29 cells after 72 h of exposure.
